# *Magnaporthe oryzae* Glycine-Rich Secretion Protein, Rbf1 Critically Participates in Pathogenicity through the Focal Formation of the Biotrophic Interfacial Complex

**DOI:** 10.1371/journal.ppat.1005921

**Published:** 2016-10-06

**Authors:** Takeshi Nishimura, Susumu Mochizuki, Naoko Ishii-Minami, Yukiko Fujisawa, Yoshihiro Kawahara, Yuri Yoshida, Kazunori Okada, Sugihiro Ando, Hideo Matsumura, Ryohei Terauchi, Eiichi Minami, Yoko Nishizawa

**Affiliations:** 1 Division of Plant and Microbial Sciences, Institute of Agrobiological Sciences, NARO, Tsukuba, Ibaraki, Japan; 2 Division of Basic Research, Institute of Crop Science, NARO, Tsukuba, Ibaraki, Japan; 3 Bioinformatics Team, Advanced Analysis Center, NARO, Tsukuba, Ibaraki, Japan; 4 Biotechnology Research Center, The University of Tokyo, Bunkyo-ku, Tokyo, Japan; 5 Iwate Biotechnology Research Center, Kitakami, Iwate, Japan; Nanjing Agricultural University, CHINA

## Abstract

*Magnaporthe oryzae*, the fungus causing rice blast disease, should contend with host innate immunity to develop invasive hyphae (IH) within living host cells. However, molecular strategies to establish the biotrophic interactions are largely unknown. Here, we report the biological function of a *M*. *oryzae*-specific gene, *R* *equired-for-Focal-* *B* *IC-* *F* *ormation 1* (*RBF1*). *RBF1* expression was induced in appressoria and IH only when the fungus was inoculated to living plant tissues. Long-term successive imaging of live cell fluorescence revealed that the expression of *RBF1* was upregulated each time the fungus crossed a host cell wall. Like other symplastic effector proteins of the rice blast fungus, Rbf1 accumulated in the biotrophic interfacial complex (BIC) and was translocated into the rice cytoplasm. *RBF1*-knockout mutants (*Δrbf1*) were severely deficient in their virulence to rice leaves, but were capable of proliferating in abscisic acid-treated or salicylic acid-deficient rice plants. In rice leaves, *Δrbf1* inoculation caused necrosis and induced defense-related gene expression, which led to a higher level of diterpenoid phytoalexin accumulation than the wild-type fungus did. *Δrbf1* showed unusual differentiation of IH and dispersal of the normally BIC-focused effectors around the short primary hypha and the first bulbous cell. In the *Δrbf1*-invaded cells, symplastic effectors were still translocated into rice cells but with a lower efficiency. These data indicate that *RBF1* is a virulence gene essential for the focal BIC formation, which is critical for the rice blast fungus to suppress host immune responses.

## Author Summary

Biotrophic pathogens grow inside living host cells by secreting “effector” proteins that suppress host innate immunity. *Magnaporthe oryzae*, which causes the most serious damage to rice, and recently also to wheat, is a hemibiotrophic fungus. During the biotrophic invasion, a host membrane-rich structure called the biotrophic interfacial complex (BIC) is focally formed at the periphery of the invasive hyphae. Several effectors have been reported to accumulate in the BIC; however, its role is unknown. In this study, we identified a novel *M*. *oryzae*-specific virulence effector gene, *R* *equired-for-Focal-* *B* *IC-* *F* *ormation 1* (*RBF1*). When *RBF1* was absent, the fungus was incapable of forming the focal BIC structure. *RBF1* expression was transiently increased each time the fungus penetrated a neighboring rice cell, which is consistent with the BIC formation in each invaded cell. The *RBF1*-disrupted mutants triggered higher immune responses and showed drastically reduced pathogenicity; however, it was able to cause disease in immuno-depressed rice plants. These results indicate that the focal BIC formation is critical for suppressing host immune responses and to the virulence of *M*. *oryzae*. The mode of action of the focal BIC is unknown, but the acquisition of *RBF1* might enable *M*. *oryzae* to combat effectively against host innate immunity.

## Introduction

Biotrophic fungi colonize inside living host tissues. To facilitate the biotrophic invasion, fungal pathogens secrete proteins called effectors and modulate host physiology, including the suppression of immune responses [1–3].

*Magnaporthe oryzae* (synonym of *Pyricularia oryzae* [4]) is the fungus causing blast disease in several graminaceous crops and is highly damaging to rice worldwide [5–7]. *M*. *oryzae* is a hemibiotroph; it colonizes living host cells during the early infection stages, which is followed by the necrotrophic stage during which conidia are produced [7]. *M*. *oryzae* forms an appressorium on the plant tissue surface by a mechanism involving recognizing plant wax components as well as sensing of surface hardness and hydrophobicity [5]. The penetration peg emerges from the appressorium to pierce the host cell wall and subsequently differentiates into invasive hyphae (IH). Primary IH are thin tubular structures and differentiate into bulbous pseudohyphae, which branch inside the infected cells [8]. At this stage, the invaded cells of the susceptible host remain alive (compatible interaction), while in the resistant host, the invaded cells show hypersensitive response-induced cell death (incompatible interaction) [8,9].

Live cell imaging using fluorescent proteins has provided new insight into the events that occur during the biotrophic interaction between *M*. *oryzae* and rice. Biotrophic IH are contained in a host membrane termed the extra-invasive hyphal membrane (EIHM) [8]. Plasma membrane (PM)-localized proteins, such as LTI6B, OsCERK1, and EL5, are detected in the EIHM [10–13], indicating the relevance of EIHM to the host PM. EIHM forms a membrane cap at the tip of the primary hypha, which is later subapically positioned as the bulbous IH develop within the first invaded cells. This plant membrane-rich structure is named the biotrophic interfacial complex (BIC) [14]. In the neighboring cells, IH are again surrounded by the EIHM, and the BIC structure initially appears adjacent to the primary hyphal tips, and subsequently localizes to subapically positions [8,14]. Symplastic effectors focally accumulate in the BIC before entering the host symplast [14,15]. Fungal secretion machinery components were reported to localize adjacent to the BIC in the BIC-associated bulbous IH, and are required for efficient secretion of symplastic effectors and pathogenicity [10]. Recently, high-resolution imaging analysis of BICs demonstrated that not only host membranes but also cytosolic components are enriched in the BIC, and symplastic effectors accumulate in the BIC in a punctate form [13]. When the EIHM was labeled with the green fluorescent protein (GFP), each punctum appeared to be encircled by GFP signals, implying that the BIC is a compex of membrane vesicles that contain symplastic effectors [16]. These results strongly suggest that the BIC is the active site of translocation for symplastic effectors in the host cell. However, direct evidence showing the biological significance of BIC formation in the interaction with rice has yet to be provided.

The elucidation of molecular functions of effectors is indispensable to understand the fungal infection strategy. In an RNA-Seq analysis, ~240 genes encoding putative secretory proteins in *M*. *oryzae* were expressed during the invasion of rice cells [17]. However, the virulence functions of only a few effectors have been demonstrated in *M*. *oryzae*. Slp1 is a chitin-binding LysM protein that accumulates at the interface between the fungal cell wall and the rice PM (the extra-invasive hyphal matrix; EIHMx). In rice, chitin oligosaccharides derived from fungal cell walls induce basal resistance to *M*. *oryzae* via recognition by the receptors, CEBiP and OsCERK1, in the PM [11,18–20]. Slp1 contributes to the virulence of *M*. *oryzae* by competitively binding with the chitin oligosaccharides, which results in evasion from the chitin-triggered immune responses [12]. An avirulence effector, AvrPiz-t, plays a role in the compatible interaction when overexpressed in rice. It interacts with the rice RING E3 ubiquitin ligase APIP6 and suppresses the generation of reactive oxygen species induced by chitin and flg22, an oligopeptide derived from flagellin protein [15]. A virulence gene, *MC69*, was identified among 78 genes for putative effectors by a large-scale disruption analysis [21]. Although how *MC69* contributes to pathogenicity is unknown, the homologs of *MC69* were found in 16 other fungi, and *MC69* in the cucumber anthracnose fungus *Colletotrichum orbiculare* was also required to infect cucumber and *Nicotiana benthamiana* leaves [21]. The disruption of a single candidate gene generally causes no clear phenotypic change [21], which strongly indicates the orchestrated actions of numerous effectors to establish infection.

Studies of effectors have often been focused on the relatively small secretory proteins consisting of less than 300 amino acids [2]. To identify effector genes that play important roles during the biotrophic invasion of *M*. *oryzae*, we searched the genes that showed drastic activations *in planta* by Super-SAGE and RNA-Seq analyses [17,22]. In this study, we characterized a novel *M*. *oryzae*-specific gene, *R* *equired-for-Focal-* *B* *IC-* *F* *ormation 1* (*RBF1*; MGG_10705). *RBF1*-knockout lines lost the ability to form the focal BIC and caused an enhanced induction of host immune responses. The knockout mutant showed severely reduced virulence in rice leaves, but was capable of infecting rice plants that were immune compromised. We discuss the biological functions of Rbf1 and the importance of focal BIC formation in suppressing host immune responses.

## Results

### *RBF1* is specifically expressed in appressoria and IH in living plants

First, we screened the genes of *M*. *oryzae* that were upregulated at 24 h post inoculation (hpi) compared with at 6 hpi by Super-SAGE analysis. Among the genes, we focused on *RBF1* because it is one of the top five genes with regard to the expression levels after invasion [17] and its knockout mutant exhibited a drastic decrease in pathogenicity.

The *RBF1* in the genome of the ‘Ina86-137’ strain encodes a putative secretory protein with 658-amino acids, which is enriched with glycine (22.8%) and alanine (19.5%) residues (S1 Fig). We compared the protein sequence of ‘Ina86-137’ with those of three rice isolates of *M*. *oryzae* in the database (S1 Fig), which showed indel sequence variations. Except for the N-terminal secretion signal sequence, which was predicted by SignalP 4.0 algorithm [23] with Y-score, 0.583, the Rbf1 protein contains no other known functional motifs. An NCBI search using the BLASTP 2.3 algorithm found no proteins with sequence similarities to Rbf1 in any other kingdom or species (E-value < 10), suggesting that *RBF1* is specific to *M*. *oryzae*. A genomic DNA hybridization analysis using probe fragments derived from *RBF1* indicated that *RBF1* exists in *M*. *oryzae* rice isolates and other *M*. *oryzae* strains isolated from barley, oat, proso millet, finger millet, and Italian ryegrass (S2 Fig). However, the genomic DNA of the blast fungus strains isolated from southern crabgrass and bamboo, which are categorized in *Pyricularia* sp. [24], did not hybridize with the *RBF1* probes (S2 Fig).

**Fig 1:**
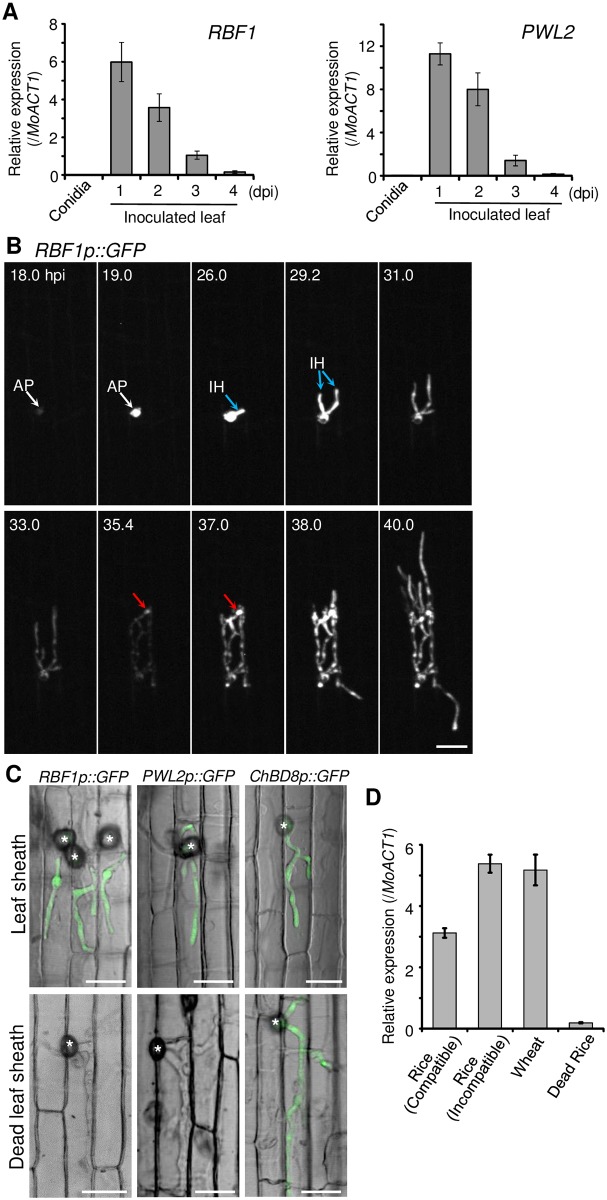
*RBF1* expression is activated when *Magnaporthe oryzae* invades living plant cells. **(A)** Quantitative RT-PCR analysis of *RBF1* and *PWL2* expression in conidia and inoculated rice leaf blades. The vertical axis indicates the amount of transcripts relative to that from the *M*. *oryzae* actin gene (*MoACT1*). Data are represented as mean values ± standard error (SE) (*n* = 3 plants). dpi, days post inoculation. **(B)** The dynamic expression of *RBF1*. *RBF1* expression during the infection process in the rice leaf sheath epidermis was monitored by a long-term time-lapse imaging method using a fungal line transformed with *RBF1p*::*GFP*. After appressoria formation, GFP signals were captured at 20-min intervals. The *z*-series of optical sections corresponding to the outer half of the inner epidermal cells were stacked to generate maximum-intensity projection images. Images displayed were selected from S1 Movie. White and blue arrows indicate the induction of *GFP* expression in the appressorium and the invasive hyphae, respectively. Red arrows indicate the re-induction of the *GFP* expression in the hyphal cell that was about to invade the neighboring cell. hpi, hours post inoculation. Bar = 20 μm. **(C)** Confocal images of *M*. *oryzae* transformants introduced with *RBF1p*::*GFP*, *PWL2p*::*GFP*, or *ChBD8p*::*GFP* growing in living rice leaf sheaths (upper) and dead rice leaf sheaths (lower) at 24 hpi. GFP images were merged with differential interference contrast images. Asterisks indicate appressoria. Bar = 20 μm. **(D)** Quantitative RT-PCR analysis of *RBF1* expression in the inoculated living leaf blades of rice and wheat, and dead rice leaf blades at 24 hpi. The vertical axis indicates the amount of transcripts relative to that from the *M*. *oryzae* actin gene (*MoACT1*). Data are represented as mean values ± SE (*n* = 4 plants).

As shown in Fig 1A, quantitative RT-PCR (qRT-PCR) confirmed that *RBF1* was highly expressed in rice leaves at 1 day post inoculation (dpi), followed by a gradual decrease for up to 4 dpi. *RBF1* expression was not detected in germinating conidia. This *RBF1* expression pattern is similar to that of *PWL2*, which encodes a known symplastic effector of *M*. *oryzae* [14] (Fig 1A).

To analyze the mode of expression of *RBF1 in planta*, we produced fungal lines transformed with *GFP* fused downstream of the promoter region of *RBF1* (*RBF1p*::*GFP*). Recently, we developed a long-term fluorescence imaging method that enables us to capture the biotrophic invasion process sequentially for over 30 h [13]. The transformant was inoculated to the inner epidermis of rice leaf sheaths, and GFP fluorescence was monitored using this successive imaging technique (Fig 1B and S1 Movie). A drastic accumulation of GFP signals was detected in the appressorium prior to penetration of the epidermal cells (18.0–19.0 hpi; white arrows in Fig 1B). The intense fluorescence was retained in the early stage of IH development (26.0–29.2 hpi; blue arrows in Fig 1B), then decreased as IH were growing in the first invaded cell (31.0–35.4 hpi). A strong re-induction of GFP expression was first observed in the top hyphal cell (35.4–37.0 hpi; red arrows in Fig 1B), which was about to penetrate into neighboring host cells, followed by a spread of the intense GFP signal to the whole IH. This gene expression pattern was detected in 16 out of 19 movies recorded (84.2%). Time-lapse imaging of a line transformed with *PWL2p*::*GFP* also showed the re-induction of the GFP signal (14 out of 29 movies: 48.3%), but the re-induction seemed to occur around the time when the hyphae penetrated into neighboring cells, which appeared later than that of *RBF1* (S2 Movie).

We also examined *RBF1* expression in the fungus inoculated to rice leaf sheaths killed by ethanol and rehydrated (see Materials and Methods). The maturation of appressoria and appressorial penetration followed by invasive growth occurred even in the dead tissues, but the expression of *RBF1* was not detected in the dead tissue (Fig 1C, left), nor was *PWL2* (Fig 1C, middle). By contrast, the expression of a *M*. *oryzae* chitinase gene, *ChBD8* (MGG_06594), which had been previously shown to be expressed in IH [25], was detected in dead, as well as in living tissue (Fig 1C, right). A qRT-PCR analysis also showed that *RBF1* was preferentially expressed in living leaf blades (Fig 1D). In addition, the expression was detected in rice leaves during both compatible and incompatible interactions, and also in wheat leaves (Fig 1D). These results indicated that the expression of *RBF1* requires interactions with living plant cells.

### Rbf1 accumulates in the BIC and is translocated into rice cells

**Fig 2:**
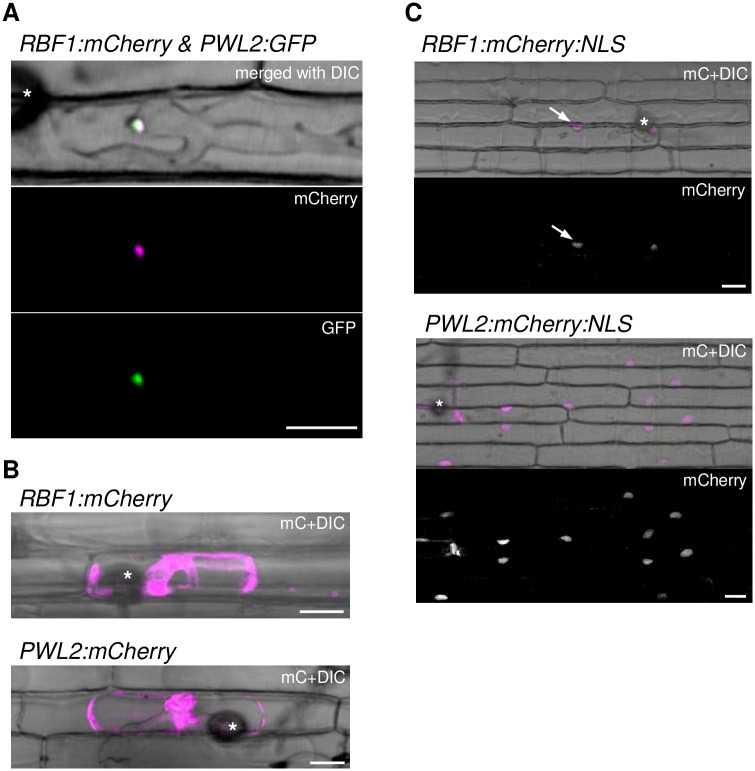
Rbf1 accumulates in the BIC and is translocated into rice cells. **(A)** Co-localization of Rbf1:mCherry with Pwl2:GFP at the BIC. Rice leaf sheaths were inoculated with *M*. *oryzae* transformed with *RBF1p*::*RBF1*:*mCherry* and *PWL2p*::*PWL2*:*GFP*, and observed using a confocal microscope at 36 hpi. DIC, differential interference contrast image. **(B)** Accumulation of Rbf1:mCherry in the rice cytoplasm. Rice leaf sheaths were inoculated with *M*. *oryzae* transformed with *RBF1p*::*RBF1*:*mCherry* and IH at 36 hpi were observed after sucrose-induced plasmolysis. Confocal mCherry images were merged with DIC images. Data obtained using a transformant with *PWL2p*::*PWL2*:*mCherry* is shown as the control. **(C)** Accumulation of the Rbf1:mCherry fused with a nuclear localization signal (NLS) in the host nucleus. Rice leaf sheaths infected by the transformant containing *RBF1p*::*RBF1*:*mCherry*:*NLS* (upper) were observed using a confocal microscope at 24 hpi. Arrows indicate rice nuclei with mCherry signals. The transformant containing *PWL2p*::*PWL2*:*mCherry*:*NLS* (lower) is shown as the control. Asterisks indicate appressoria. Bar = 10 μm.

The localization of Rbf1 in rice cells was analyzed by live-cell fluorescence imaging. We produced a *M*. *oryzae* line simultaneously expressing two fusion proteins, Rbf1:mCherry and Pwl2:GFP, each driven by its own promoter. After inoculating the leaf sheaths with the transformant, fluorescent signals were observed. Rbf1:mCherry complimented Rbf1 function, as described later. Pwl2:GFP marks the BIC [14]. As shown in Fig 2A, a concentrated mCherry signal was detected, which largely overlapped the GFP signal. Rbf1:mCherry accumulation was also detected in the BIC at the tip of the primary IH (S3A Fig).

Rbf1:mCherry was detected in the cytoplasm of rice cells where plasmolysis was induced (Fig 2B). Moreover, the fluorescent signal of Rbf1:mCherry, when fused with the nuclear localization signal of SV40 (Rbf1:mCherry:NLS), was detected in the host nucleus in addition to the BIC (Fig 2C upper panels). While Pwl2:mCherry:NLS was detected in the uninvaded neighboring cells in addition to the first invaded cell (Fig 2C lower panels), as already reported [14], the signals for Rbf1 were exclusively detected in the invaded cells.

Rbf1 contains a putative secretion signal at the N-terminus. To examine the function of the signal sequence, we produced the *M*. *oryzae* lines with *RBF1p*::*RBF1ΔSS*:*mCherry*, encoding mCherry fused with an Rbf1 that is lacking the signal sequence, and with *RBF1p*::*SS*:*mCherry*, encoding mCherry fused with the signal sequence at the N-terminus. Observations of rice leaf sheaths inoculated with these transformants revealed that the deletion of the signal sequence resulted in the accumulation of the fluorescence signal in IH (S3B Fig), and the attachment of the signal sequence to mCherry led to its localization to the BIC (S3C Fig). These results indicated that the BIC localization of Rbf1 requires the signal sequence but not the mature form of Rbf1.

### The *RBF1*-disrupted fungus shows a drastic defect in pathogenicity

To investigate *RBF1* function, we produced the *RBF1*-disrupted mutant (*Δrbf1-1*) by homologous recombination using a GFP knock-in binary vector [25]. A genomic DNA hybridization analysis confirmed that the *RBF1* coding region was replaced with *GFP* and the hygromycin resistance gene; thus, *Δrbf1-1* expressed *GFP* under the *RBF1* promoter (S4 Fig). The knockout mutant (KO) showed normal growth and sporulation on an agar medium (S5A and S5B Fig). Additionally, the KO was indistinguishable from its parental wild-type strain (WT) in the morphologies of conidia and appressoria (S5C Fig), and in the development of appressoria on glass plates (S5D Fig).

**Fig 3:**
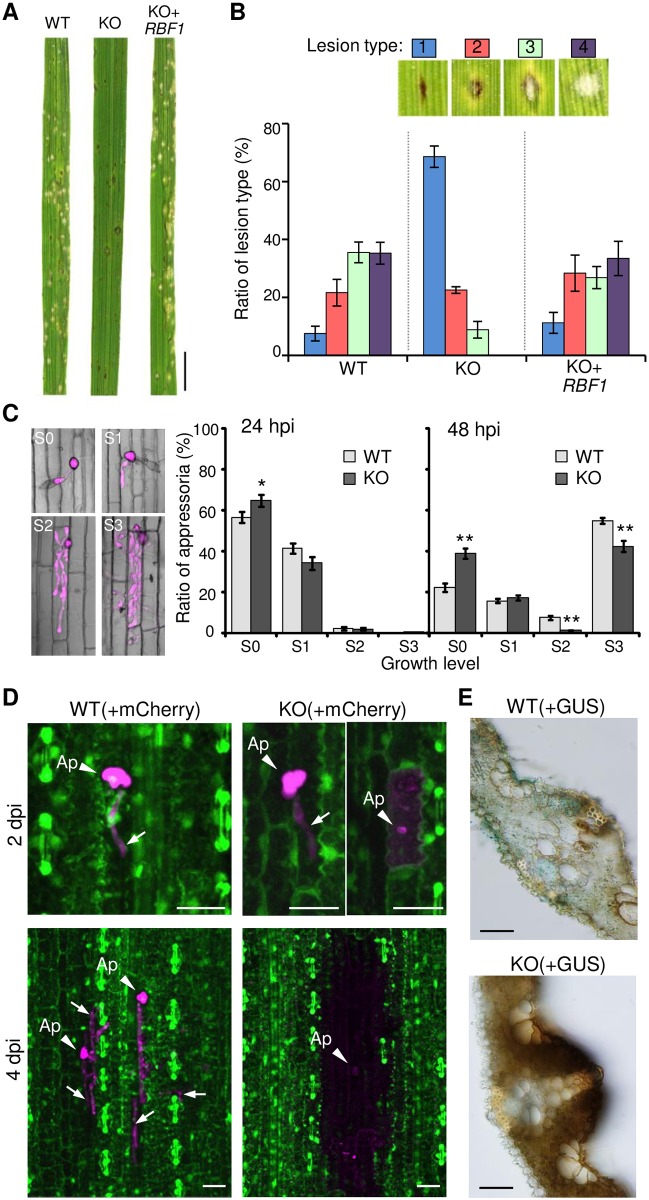
*RBF1* is a virulence determinant in *Magnaporthe oryzae*. **(A)** Representative symptoms on the 6^th^ leaf blades at 5 days after inoculation. Rice plants were sprayed with a conidial suspension of the wild-type strain (WT), an *RBF1*-knockout line (*Δrbf1-1*; KO), and a gene complementation line (KO*+RBF1*). Bar = 5 mm. **(B)** Evaluation of lesion types in leaf blades. Lesions formed at 5 days after spray-inoculation were counted according to the classifications displayed. Data are represented as the mean percentages ± SE (*n* = 5 plants). **(C)** Comparison of the development of invasive hyphae in rice leaf sheaths between WT and *Δrbf1-1* (KO). Infection levels in leaf sheaths were assessed for each appressorium under a microscope and categorized as no invasion (S0), short invasive hyphae in one cell (S1), highly-branched invasive hyphae in one cell (S2), and multiple cell invasion (S3). To illustrate each category, typical images using a WT line transformed with *TEFp*::*mCherry* are displayed. Data are represented as the mean percentages ± SE [*n* = 14 plants (24 h post inoculation; hpi) and 23 plants (42 hpi)]. Student’s *t*-test was performed on arcsine-transformed data between WT and KO (*, *P* < 0.05; **, *P* < 0.005). The total numbers of appressoria observed per line were ~ 1,500 (24 hpi) and 3,000 (48 hpi). **(D)** Confocal images of rice epidermal cells in inoculated leaf blades. Transgenic rice plants constitutively expressing *GFP* under the CaMV *35S* promoter were inoculated with the WT (left) or *Δrbf1-2* line (KO; right) transformed with *TEFp*::*mCherry*. GFP and mCherry signals were merged. Note that the disappearance of the GFP signal (green) indicates host cell death. Arrows indicate invasive hyphae. Ap, appressorium. Bar = 20 μm. **(E)** Transverse sections of inoculated rice leaf blades at 6 dpi. To visualize invasive hyphae, the WT (upper) and *Δrbf1-1* (KO; lower) were transformed with *TEFp*::*GUS*. Spot-inoculated rice leaf blades were stained for β-glucuronidase activity, hand-sectioned, and observed by light microscopy. Bar = 0.1 mm.

Next, we assayed the virulence of the KO. When intact rice plants were sprayed with a conidial suspension of the WT, acute susceptible lesions (white-gray spots without browning) were formed at 5 dpi (Fig 3A). However, the KO showed severely impaired virulence in rice leaves, and this phenotype was complemented by a genomic DNA fragment carrying *RBF1* (Fig 3A). Although the number of lesions per unit area was comparable between the WT and KO (S6 Fig), the KO did not form acute susceptible lesions, but resistant lesions (small brown specks) in leaf blades (Fig 3B). The serious defect in virulence was also shown in the spot-inoculation assay used to evaluate pathogenicity hereafter (S7 Fig).

We observed fungal invasion during early infection stages using a leaf sheath inoculation method followed by microscopic observations. As shown in Fig 3C, The KO was able to penetrate rice epidermal cells although the rate was significantly lower than that of the WT. The KO also showed significantly lowered rates of hyphal development and colonization at 48 hpi (Fig 3C).

To visualize the mode of infection in leaf blades, we inoculated leaves of a transgenic rice plant constitutively expressing GFP (*35S*::*GFP* rice) with the WT or KO line that constitutively expressed mCherry in the cytosol. For this assay, we generated a new KO mutant (*Δrbf1-2*) that did not contain *GFP* (S8 Fig). As shown in Fig 3D, at 2 dpi, the WT successfully invaded rice cells, and GFP signals were detected in the invaded host cell. Some infection sites in the KO-inoculated leaf blades showed a similar fluorescence pattern to that of the WT-invaded cells, but other sites showed the spread of mCherry signals over the invaded epidermal cell, indicating fungal cell lysis. At 4 dpi, both mCherry and GFP signals in the neighboring two or three cell layers, as well as in the first invaded cells, diminished in the KO-inoculated leaves, whereas WT developed the IH toward the flanking cells, and the GFP signals around the infection site were maintained. Moreover, transverse sections of leaf blades inoculated with a KO line showed an intense browning compared with the lesions formed in the WT-inoculated leaf blades (Fig 3E and S9 Fig). These results showed that the KO triggers host cell death accompanied by browning.

### Phytoalexin (PA) production is more activated in rice leaves inoculated with the *RBF1*-disrupted fungus

**Fig 4:**
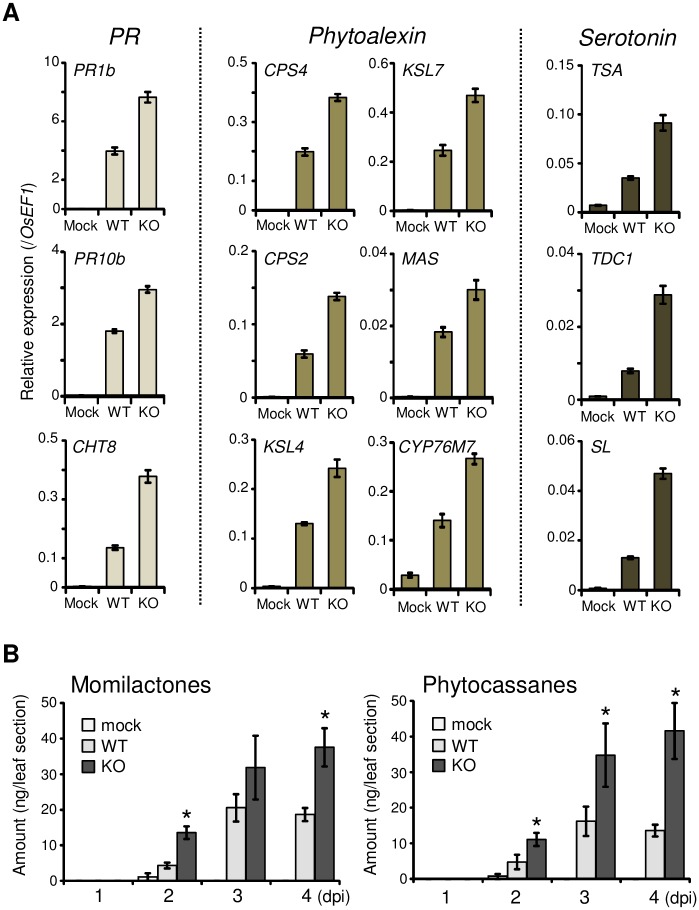
*RBF1* is required to suppress the activation of host immune responses. **(A)** Enhanced activation of rice defense-related genes by KO. Rice leaf blades were spotted with a conidial suspension of the WT fungus or *Δrbf1-1* (KO). RNA was extracted from the inoculated leaves at 2 dpi and subjected to qRT-PCR analysis. The vertical axis indicates the amount of transcripts relative to that from rice *eEF-1α* (*OsEF1*). Bars represent the mean values ± SE (*n* = 4 plants). *PR*, pathogenesis-related genes; Mock, spotted with water. **(B)** Enhanced accumulation of rice diterpenoid phytoalexins by KO. Momilactones and phytocassanes in inoculated leaf blades were quantified using an HPLC-MS/MS spectrometer. Data from five to seven independent extracts of two inoculation assays are represented as mean values ± SE. Asterisks indicate significant differences compared with the WT data (Student’s *t*-test, *P* < 0.05).

To examine whether the KO is defective in suppressing host immune responses, we compared the expression levels of rice genes that exhibited infection-specific expression at 2 dpi in KO-inoculated and WT-inoculated rice leaves using an RNA-Seq analysis. We identified 106 genes that were expressed at least twofold higher in KO-inoculated leaves than in WT-inoculated leaves (S1 Table). They included 11 pathogenesis-related genes (*PR*) and 10 genes encoding enzymes for diterpenoid PA synthesis. The expression of genes involved in serotonin synthesis was also more highly induced in KO-inoculated leaves than in WT-inoculated leaves. The upregulation of a subset of these defense-related genes was further confirmed by qRT-PCR (Fig 4A).

We measured PA amounts in the inoculated leaves using HPLC-MS/MS (Fig 4B). Consistent with the gene expression, the accumulation of diterpenoid PAs was detected at 2 dpi both in WT- and KO-inoculated leaves. For up to 4 dpi, the levels of both momilactones and phytocassanes were higher in KO- than WT-inoculated leaves. In contrast, the induction levels of *NOMT*, which encodes the key enzyme for the synthesis of a flavonoid PA, sakuranetin [26], were similar between WT- and KO-inoculated leaves (S10A Fig). Sakuranetin accumulated in KO-inoculated leaves at slightly, but not significantly, lower levels than those in WT-inoculated leaves (*P* > 0.1; S10B Fig). Thus, Rbf1 is required to suppress the expression of a specific subset of defense-related genes, which results in the reduced levels of diterpenoid PAs upon infection.

### The *RBF1*-disrupted fungus infects immuno-depressed rice plants

**Fig 5:**
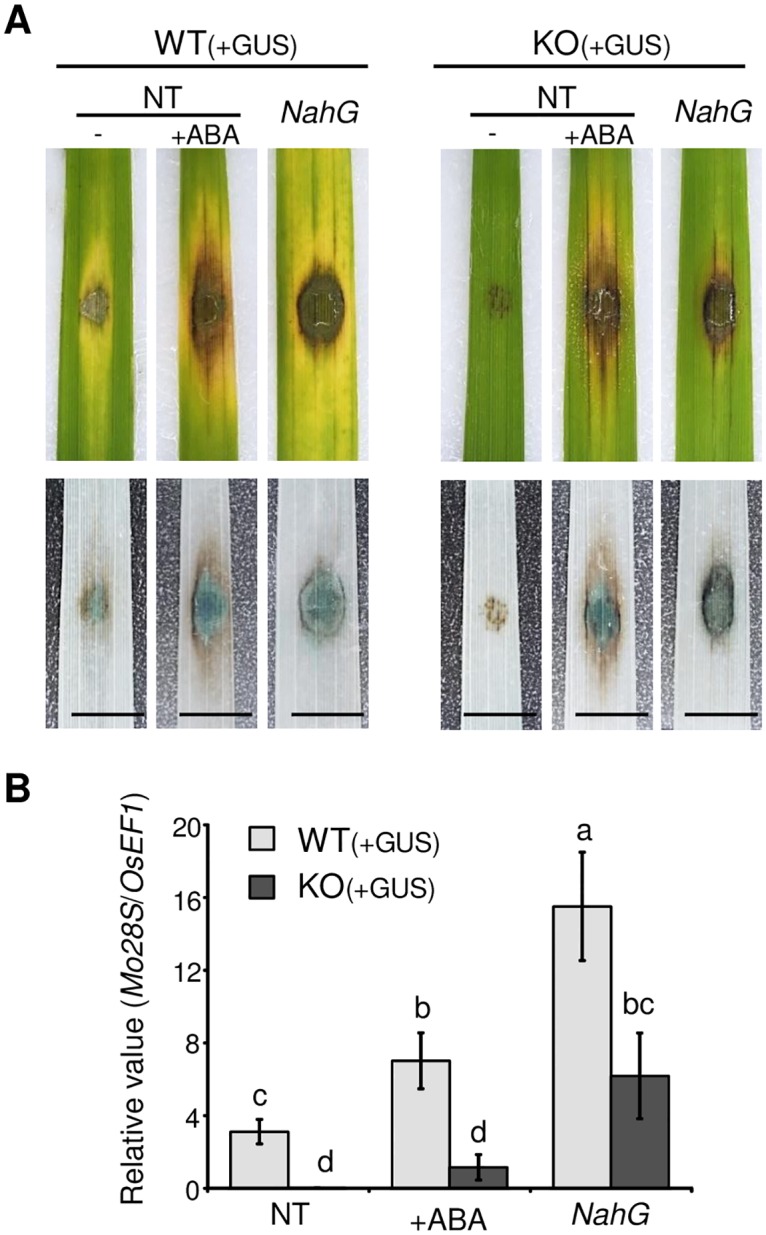
The *RBF1*-disrupted blast fungus infects rice plants with impaired immunity. **(A)** Symptoms on the spot-inoculated rice leaf blades at 5 dpi (upper) and the GUS staining (lower). Both the WT and *Δrbf1-1* (KO) were transformed with *TEFp*::*GUS* to visualize invasive hyphae. NT, non-transgenic rice; +ABA, inoculated with 30 μM abscisic acid; *NahG*, transgenic rice expressing the salicylate hydroxylase gene. Bar = 5 mm. **(B)** Proliferation of *M*. *oryzae* in leaf blades at 6 dpi evaluated by quantitative PCR. DNA amount of *M*. *oryzae 28SrDNA* (*Mo28S*) relative to rice *eEF-1α* (*OsEF1*) in spot-inoculated leaf fragments were measured. Data are represented as mean values ± SE (*n* = 7 plants for NT and +ABA, and *n* = 5 plants for *NahG* samples). Different letters above bars indicate significant differences at *P* < 0.05 (Student’s *t*-test of paired comparison).

Based on the above data, the KO was hypothesized to be able to infect plants in which the elicitation of immune responses is suppressed. In higher plants, including rice, salicylic acid (SA) is involved in immunity, as supported by the observation that transgenic plants expressing *NahG*, a bacterial SA-inactivating gene, show depressed disease resistance [27]. The action of SA is antagonized by abscisic acid (ABA) [27–30]. In fact, the activation of most of the *M*. *oryzae*-responsive genes tested was drastically suppressed by an ABA treatment or *NahG* expression at 2 dpi (S11 Fig). Thus, we tested the virulence of KO in ABA-treated or *NahG*-expressing rice plants. As a result, the KO caused compatible-type disease symptoms (Fig 5A). Measurements of fungal biomasses also confirmed the proliferation of the KO in ABA-treated or *NahG*-expressing leaves although the effect of ABA-treatment on the KO infection was not statistically significant (Fig 5B). These results further supported the hypothesis that *RBF1* is critical to suppressing host immunity.

### The *RBF1*-disrupted fungus shows dispersed BIC formation

To further analyze the invasive growth of the KO, we transformed WT and *Δrbf1-1* with *BAS4p*::*BAS4*:*mCherry* and compared the fluorescence patterns in the rice leaf sheaths inoculated with the transformants. Bas4 is an apoplastic effector accumulating significantly in the EIHMx and also at the BIC [13,14]. As a result, the mCherry signals outlining the mutant IH appeared normal, but, the intense mCherry signals that should be at the BIC position were diffused around the IH of the KO transformant (S12A Fig; captured image data *n* = 33).

**Fig 6:**
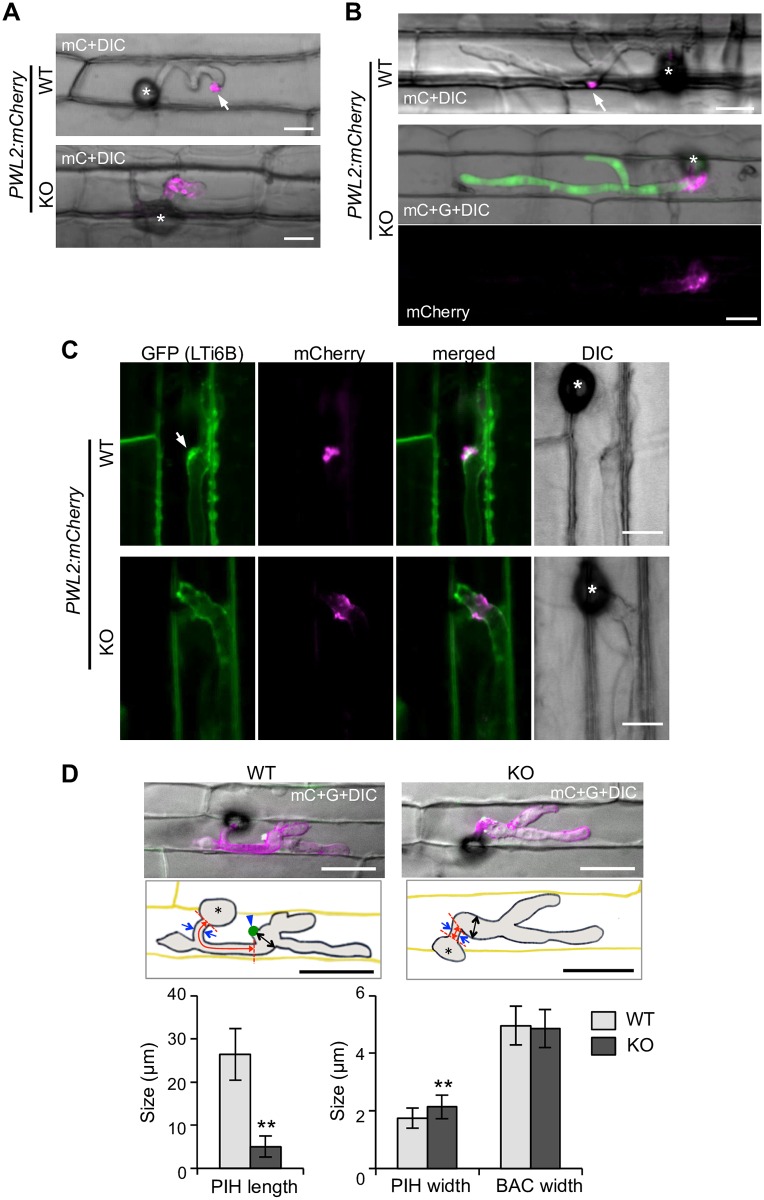
Rbf1 is required for the focal BIC formation and normal hyphal development. Confocal images of rice leaf sheath cells infected by the WT or KO line harboring *PWL2p*::*PWL2*:*mCherry* at 24 hpi **(A)** and 36 hpi. **(B)**. Note that the coding region of *RBF1* in the genome was replaced with *GFP* in the *Δrbf1-1* used (KO), thus the KO-based transformants express free GFP (green) driven by the *RBF1* promoter. Bar = 10 μm. **(C)** Confocal images of the extra-invasive hyphal membranes (EIHM) and a BIC-localizing effector protein at 30 hpi. Rice leaf sheaths transformed with *35S*::*GFP*:*LTI6b* were inoculated with the WT or KO line harboring *PWL2p*::*PWL2*:*mCherry*. Arrow indicates the aggregation of EIHM at the BIC position. Note that the KO-invaded rice cell shows the broad distribution of the BIC marker effector around the IH and no accumulation of the GFP signals at the mCherry signals. Bar = 10 μm. **(D)** Comparison of invasive hyphal shape. Rice leaf sheaths were inoculated with the WT or *Δrbf1-2* (KO) line harboring both *PWL2p*::*PWL2*:*GFP* and *BAS4p*::*BAS4*:*mCherry* and observed using a confocal microscope at 30 hpi. The *z*-series of optical sections were stacked to generate maximum-intensity projection images. Confocal images of the representative infection sites are shown with illustrations indicating the hyphal parts measured. Red, blue, and black arrows indicate the length and width of the primary IH (PIH), and the width of the BIC-associated cell (BAC), respectively. Arrowhead indicates the BIC. Bar = 20 μm. Data of the IH sizes measured using ImageJ (http://imagej.nih.gov/ij) are represented as mean values ± standard deviation (*n* = 57 infection sites). Asterisks above bars indicate significant differences compared with the WT data (Student’s *t*-test, *P* < 0.01). DIC, differential interference contrast image; mC, mCherry image; G, GFP image. Asterisks, appressoria.

Thus, we observed BICs using fungal lines transformed with *PWL2p*::*PWL2*:*mCherry*. The WT-based transformant showed a focal accumulation of Pwl2:mCherry in a typical BIC (upper panels in Fig 6A and 6B) as reported previously [14]. By contrast, host cells invaded by the KO-based transformant showed dispersal of the normally BIC-focused mCherry signals around the primary and the first bulbous IH (lower panels in Fig 6A and 6B; *n* > 50). The mCherry signals were often observed as puncta distributed along the IH, and the normal focal BICs were never detected in KO-invaded rice cells. Another putative symplastic effector protein (MGG_10010; S13A Fig) fused with mCherry also showed a broad accumulation in the KO-invaded cells (S13B Fig; *n* = 6). Further analysis of the effector localization at BICs using transformants containing both *PWL2p*::*PWL2*:*GFP* and *BAS4p*::*BAS4*:*mCherry* also revealed that the KO-based transformant showed a dispersed accumulation of both effectors around the IH (*n* > 50), whereas the WT-based transformant showed the focal accumulation of Pwl2 and Bas4 at one place (S14 Fig).

We further analyzed BIC structures in KO-invaded cells using transgenic rice plants expressing a PM/EIHM-marker protein, GFP:LTI6B [31,32]. In WT-invaded cells, the GFP signals aggregated at the mCherry signals from Pwl2:mCherry (arrow in Fig 6C) or Bas4:mCherry (S12B Fig) to form dome-shaped BIC structures, in addition to outlining the IH, as reported previously [10,13]. By contrast, KO-invaded cells showed the diffused GFP signals along the IH in association with altered accumulation patterns of Pwl2:mCherry (*n* = 20) and Bas4:mCherry (*n* = 5) (lower panels in Fig 6C and S12B Fig). Observations of host cytosol using *35S*::*GFP* rice also demonstrated that the KO-invaded cells lacked the typical dome-shaped BIC structures and showed the dispersed localization of effector proteins along the IH (S15 Fig). These results indicated that the lack of *RBF1* caused not only dispersal of the normally BIC-focused effector localization but also the impaired aggregation of the EIHM.

### The *RBF1*-disrupted fungus is defective in IH differentiation

In addition to the defective BIC formation, IH shape appeared abnormal in the KO. The WT-based lines developed the thin tubular IH with the focal BIC at the tip, which then differentiated into the bulbous cell (upper panels in Fig 6A and 6B). By contrast, the KO-based lines formed thick IH shortly after invasion (lower panels in Fig 6A and 6B). Measurement of the length of the primary IH (the distance between the appressorium and the BIC-associated first bulbous hypha) showed that the KO formed ca. five times shorter primary IH than that of the WT (Fig 6D). Comparison of the width of the primary IH showed that the KO formed significantly thicker primary IH than the WT, but the thickness of the first bulbous hyphae, normally the focal BIC-associated cell, was comparable (Fig 6D).

### *RBF1* contributes to virulence through focal BIC formation

**Fig 7:**
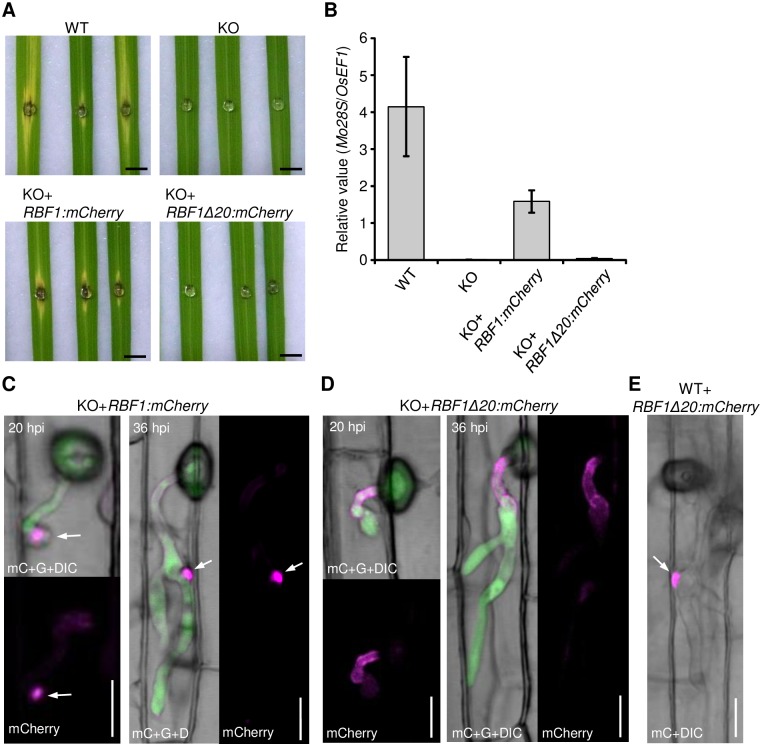
Focal BIC formation correlates with the virulence of *Magnaporthe oryzae*. **(A)** Symptoms on the spot-inoculated rice leaf blades at 6 dpi. The *Δrbf1-1* (KO) was further transformed with *RBF1p*:*RBF1*:*mCherry* or *RBF1p*::*RBF1Δ20*:*mCherry*. Rbf1Δ20:mCherry has a 20 amino acid-deletion (corresponding to Pro^320^-Gly^339^). Bar = 5 mm. **(B)** Proliferation of *M*. *oryzae* in leaf blades at 6 dpi evaluated by quantitative PCR. DNA amount of *M*. *oryzae 28SrDNA* (*Mo28S*) relative to rice *eEF-1α* (*OsEF1*) in spot-inoculated leaf fragments were measured. Data are represented as mean values ± SE (*n* = 4 plants). Confocal images of leaf sheath cells invaded by the *Δrbf1-1* lines (KO) transformed with *RBF1p*::*RBF1*:*mCherry*. **(C)** or *RBF1p*::*RBF1Δ20*:*mCherry*. **(D)** Both transformants express GFP (green) owing to the replacement of the coding region of the endogenous *RBF1* with *GFP* in *Δrbf1-1*. Arrows indicate the focal localization of Rbf1:mCherry in the BIC. Bar = 10 μm. **(E)** Confocal image of a leaf sheath cell invaded by the WT line transformed with *RBF1p*::*RBF1Δ20*:*mCherry* at 36 hpi. Bar = 10 μm.

We obtained unexpectedly an *RBF1* mutant, *RBF1Δ20*, which has a 60 bp-deletion (corresponding to Pro^320^-Gly^339^). The introduction of *RBF1p*::*RBF1*:*mCherry* into *Δrbf1-1* largely compensated for the impaired pathogenicity, whereas *RBF1p*::*RBF1Δ20*:*mCherry* did not (Fig 7A and 7B), indicating that Rbf1:mCherry, but not Rbf1Δ20:mCherry, was functional. We used these lines to clarify the relationship between the defect in pathogenicity and BIC formation in the KO. We observed the BICs using the fluorescence from Rbf1:mCherry at different time points. In the rice cells infected by the complemented line, Rbf1:mCherry was found at the tip of the primary IH at 20 hpi (*n* = 3), and then, beside the first bulbous IH at 36 hpi (*n* = 40) (Fig 7C), which was similar to the process observed in the cells invaded by the WT line harboring *RBF1p*::*RBF1*:*mCherry* (S3A Fig and Fig 2A). These results indicated that Rbf1:mCherry complements the KO to form focal BICs. The short primary IH phenotype also appeared to be canceled by Rbf1:mCherry. By contrast, *RBF1p*::*RBF1Δ20*:*mCherry* could not recover the defect in the focal BIC formation in KO (Fig 7D; *n* > 35). Rbf1Δ20:mCherry was confirmed to accumulate focally in the predicted BIC in the WT background (Fig 7E).

**Fig 8:**
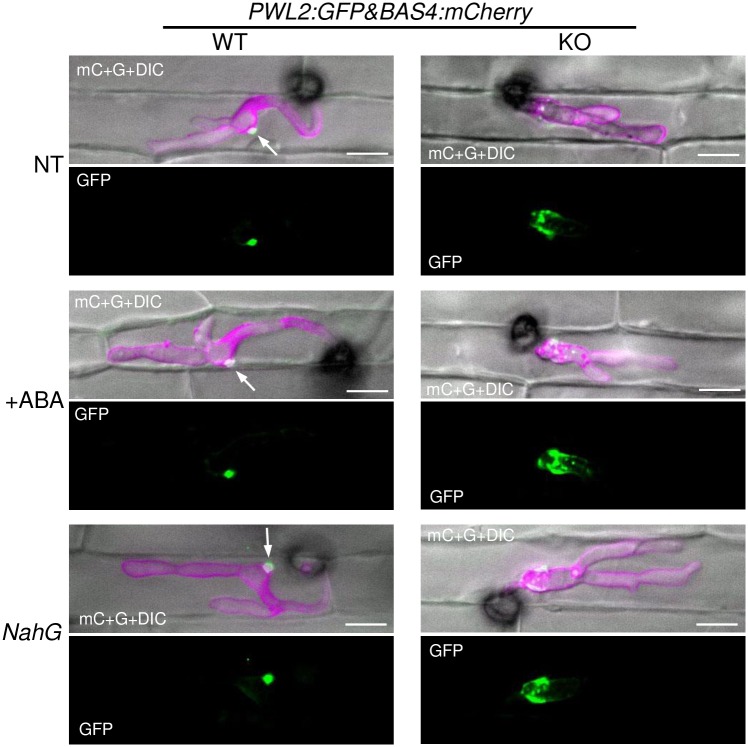
Host immunity does not affect the dispersed BIC formation in the *RBF1*-disrupted fungus. Rice leaf sheaths were inoculated with the WT or *Δrbf1-2* line transformed with both *PWL2p*::*PWL2*:*GFP* and *BAS4p*::*BAS4*:*mCherry* and observed using a confocal microscope at 30 hpi. The *z*-series of optical sections were stacked to generate maximum-intensity projection images. NT, non-transgenic rice; +ABA, inoculated with 30 μM abscisic acid; *NahG*, transgenic rice expressing the salicylate hydroxylase gene. Bar = 10 μm.

To clarify whether the defect in the focal BIC formation caused a reduction in virulence, or the higher host defense responses triggered by the KO affected the establishment of the focal BIC, we analyzed BICs in the rice plants that were immune compromised. As a result, in contrast to the WT-based transformant, which showed the focal accumulation of Pwl2:GFP at one place, the KO-based transformant showed the dispersed puncta of Pwl2:GFP signals even in the ABA-treated cells (*n* = 20) and *NahG*-expressing cells (*n* = 33) (Fig 8). The short primary IH phenotype also appeared unchanged in these cells.

### Translocation of Pwl2 remains, but decreases in the absence of *RBF1*

**Fig 9:**
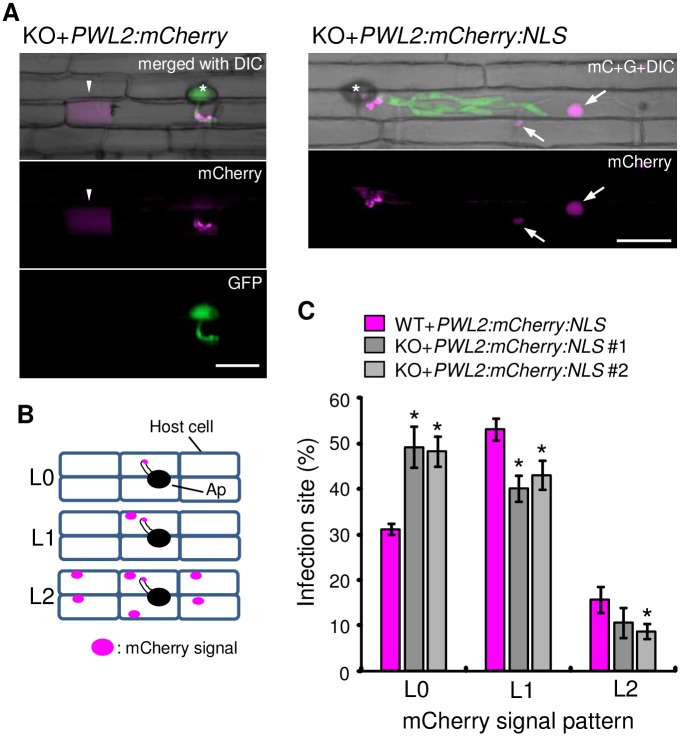
Translocation levels of a symplastic effector are impaired by the *RBF1* disruption. **(A)** Confocal images of rice leaf sheath cells invaded by the *Δrbf1-1* (KO) lines containing *PWL2p*::*PWL2*:*mCherry* (left) or *PWL2p*::*PWL2*:*mCherry*:*NLS* (right) at 24 hpi. Arrowheads and arrows indicate host cytoplasm and nuclei with mCherry signals (red), respectively. Asterisks, appressoria. Bar = 20 μm. **(B)** Categories of the mCherry signal pattern. L0, no mCherry signals in the host nucleus; L1, mCherry signals only in the nucleus of the invaded cell; L2, mCherry signals in the first invaded cell and the neighboring uninvaded cells. **(C)** A lack of *RBF1* causes a reduction in the spread of Pwl2. Rice leaf sheaths inoculated with the WT or KO line containing *PWL2p*::*PWL2*:*mCherry*:*NLS* were observed at 24 hpi, and mCherry signal patterns were classified into the three categories illustrated in **(B)**. Data are represented as the mean percentages ± SE [*n* = 4 tests (WT) and 5 tests (KO)]. Asterisks above the bars indicate significant differences compared with the data of the WT line (*P* < 0.05, Student’s *t*-test on arcsine-transformed data).

It has been proposed that symplastic effectors are translocated into host cells through the BIC after being secreted from IH [10,14]. Thus, we examined the effector translocation in KO-invaded rice cells using the *Δrbf1-1* lines containing *PWL2p*::*PWL2*:*mCherry* or *PWL2p*::*PWL2*:*mCherry*:*NLS*. In the cell invaded by the KO expressing Pwl2:mCherry, the mCherry signal was detected in the host cytosol in addition to around the primary IH (left panels in Fig 9A). The GFP expressed by the *RBF1* promoter was exclusively detected in the fungal body, indicating that the accumulation of Pwl2:mCherry in the host cytosol was not a result of fungal lysis. In the cell invaded by the KO expressing Pwl2:mCherry:NLS, the mCherry signals were detected in the host nuclei in addition to the region around the primary IH (right panels in Fig 9A). These results indicated that Pwl2 was translocated into the host cytoplasm despite the irregular BIC morphology.

Next, we assessed the effector translocation in KO-invaded cells by comparing the spread level of Pwl2 with that of the WT. Rice leaf sheaths inoculated with transformants harboring *PWL2p*::*PWL2*:*mCherry*:*NLS* were observed at 24 hpi, when the IH was still in the first invaded cells. The patterns of mCherry-positive nuclei at each infection site were divided into three categories: L0 (no mCherry signal was found in the nuclei: no translocation), L1 (mCherry-positive nucleus only found in the first invaded cell), and L2 (neighboring cells also contained the mCherry signal) (Fig 9B). As a result, the ratio of L0 in KO-invaded cells was significantly higher, while that of L1 and L2 were lower, than that in WT-invaded cells (Fig 9C). There was no significant difference in the expression level of *PWL2*:*mCherry* among the two independent KO-based and a WT-based transformants used (S16 Fig).

We further analyzed the effector translocation using an avirulence gene, *AVR-Pik*, in the KO. *AVR-Pik* encodes a symplastic effector that causes hypersensitive cell death in rice cells carrying a resistance gene, *Pik* [33]. We inoculated rice leaf sheaths of the resistant cultivar (Nipponbare Kanto-BL5) with the WT or KO line harboring *TEFp*::*mCherry* and counted the infection sites that showed the mCherry leakage to the invaded host cell under a microscope at 30 hpi. The mCherry leakage indicates IH lysis [13]. As a result, the ratio of IH lysis in the KO-invaded rice cells was lower than that in the WT-invaded cells (S17A Fig). The incompatible interaction was not visibly altered when the rice leaf blades were spray-inoculated with the KO (S17B Fig).

## Discussion

### Characteristic mode of *RBF1* expression during infection

In this study, we identified a novel virulence gene, *RBF1*. The expression of *RBF1* showed a drastic induction after invasion in qRT-PCR analysis (Fig 1A). A long-term live cell imaging method revealed that the *RBF1* expression is repeatedly activated prior to the invasion of each host cell (Fig 1B and S1 Movie), which is consistent with the BIC formation in each invaded host cell [14]. It is unknown at the moment whether this expression pattern with two successive waves is specific to *RBF1*. The long-term live cell imaging indicated the possibility that the expression level of *PWL2* also changes during the infection process (S2 Movie), implying that the re-induction of gene expression is common in effector proteins. *M*. *oryzae* developed appressoria and penetrated into dead leaf tissue to form IH. The expression of *RBF1* and *PWL2* was only detected in the appressoria and IH that were formed in the living tissue (Fig 1C). Therefore, the infection stage-specific expression of *RBF1* and *PWL2* may require signals generated during the biotic interactions with plants. Recently, the global profiling of gene expression showed that transcription factors in *M*. *oryzae* change their expression levels upon contact with host plants [34]. Several Zn_2_Cys_6_ fungal-specific transcription factors are involved in virulence of *M*. *oryzae* [35]. However, the regulation mechanism of effector gene expression is largely unknown. Because *RBF1* was critical in promoting the virulence of *M*. *oryzae*, elucidating the molecular basis of *RBF1* expression would provide us with a potential strategy to control rice blast disease.

### *RBF1* is critically involved in virulence of *M*. *oryzae*

In the absence of *RBF1*, proliferation in rice leaves was severely restricted, and host cell death was induced during the early infection stage (Fig 3 and S7 Fig). A global gene expression analysis and the quantification of PA in the infected rice leaves demonstrated that the lack of *RBF1* causes the enhanced activation of host immune responses although not all the expression of *M*. *oryzae*-responsive genes was affected (Fig 4 and S1 Table). Of two groups of PAs, inoculation with *Δrbf1* elevated the levels of diterpenoid but not flavonoid PAs (Fig 4B and S10 Fig). The rapid accumulation of diterpenoid phytoalexins associated with hypersensitive response-induced cell death is a hallmark of rice plants exhibiting resistance to restrict the growth of *M*. *oryzae* [36]. A rice mutant with a defect in *OsCPS4* expression accumulates a lower level of momilactone A upon fungal infection and shows enhanced susceptibility to *M*. *oryzae* [37]. Furthermore, in *Δrbf1*-inoculated rice leaves, the three genes for serotonin biosynthesis, i.e., tryptophan synthase, tryptophan decarboxylase, and tryptamine 5-hydroxylase, were upregulated (Fig 4A), suggesting the enhanced generation of serotonin. In fact, inoculation with *Δrbf1* led to the increased accumulation of brown material in rice leaf tissues (Fig 3). Serotonin was reported to accumulate mainly in the cell walls within the lesion formed by *M*. *oryzae* or *Bipolaris oryzae*, and its deficient *sl* rice forms non-browning lesions (the Sekiguchi lesions) after inoculation and shows increased susceptibility to these fungal pathogens [38]. Therefore, it is very likely that the enhanced accumulation of diterpenoid PAs and serotonin was a cause of the arrest of fungal proliferation in *Δrbf1*-inoculated leaves.

The lesion formation and proliferation of *Δrbf1* were partially restored in transgenic rice leaves with lowered levels of SA or in leaves treated with ABA, an antagonist of SA (Fig 5). In these plants, the expression levels of genes involved in the biosynthesis of PAs and serotonin, in addition to the *PR* genes, was severely diminished (S11 Fig). Taken together, our data strongly suggest that Rbf1 is a virulence effector critical for the suppression of host immunity.

### Rbf1 is required for the focal BIC formation

The live cell imaging of BICs revealed that not only the localization of effector proteins, but also the focal aggregation of EIHM and host cytosol, was disintegrated in *Δrbf1*-invaded cells (Fig 6C and S12B and S15 Figs). Moreover, the disruption of *RBF1* caused the abnormal IH shape; the length of the normally thin tubular primary hypha was significantly shorter and thicker in *Δrbf1* compared to the WT (Fig 6A and 6D). Because the dispersed BIC and the short primary IH phenotypes were not canceled in the rice plants with artificially depressed immune responses (Fig 8), the phenotypes are considered not to be a secondary effect of increased host immune responses resulting from the *RBF1* defect.

**Fig 10:**
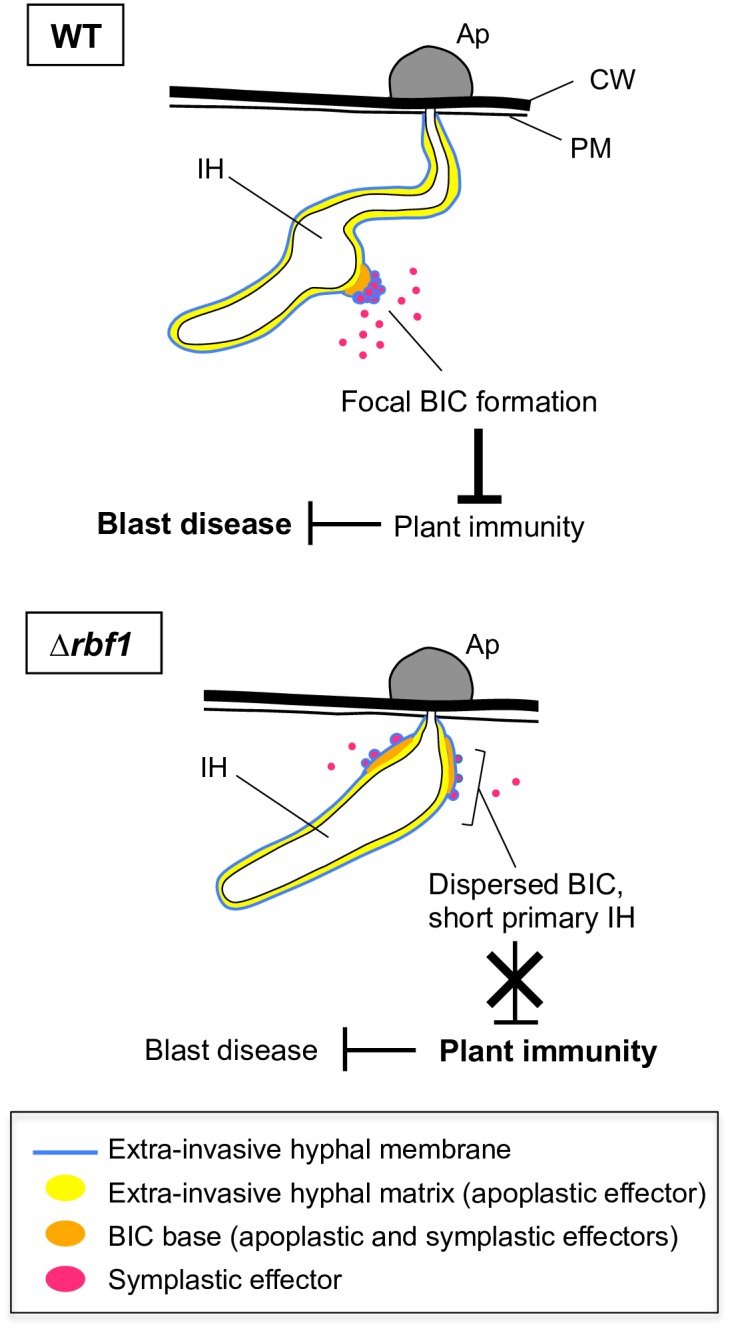
Summary of phenotypes shown in the wild-type *Magnaporthe oryzae* and the *RBF1*-disrupted mutant. The biotrophic interfacial complex (BIC) is a specialized region of the EIHMx focally formed at the tip of the tubular invasive hypha that differentiates into the bulbous pseudohyphae. The BIC comprises the aggregated EIHM in which symplastic effectors detected as a cluster of puncta and the BIC base in which apoplastic effectors also preferentially accumulate. From the characterization of the knockout mutants (Δ*rbf1*), it is deduced that Rbf1 plays a crucial role in the development of the focal BIC structure and the hyphal differentiation, which is required to lower the activation of host immune response, thus allowing the establishment of the biotrophic invasion. Ap, appressorium; CW, host cell wall; IH, invasive hyphae; PM, host plasma membrane.

High-resolution imaging of BICs suggests that the BIC is composed of two regions: one containing both apoplastic and symplastic effectors (the BIC base) and the other containing only symplastic effectors, which is detected as a cluster of puncta [13]. In *Δrbf1*-invaded cells, the Bas4 localization outlining the IH appeared normal, but its accumulation that should be normally at the BIC base was diffused (S12 and S15 Figs). These observations imply that Rbf1 is indispensable to organize the focal BIC base (Fig 10), which is consistent with the localization of Rbf1 at the BIC (Figs 2A and 7C and S3A Fig).

The BIC is a specific EIHMx region, which is proposed to play a role in the translocation of symplastic effectors [10,14]. The secretion of effector proteins toward the BIC is regulated by two exocyst components, Exo70 and Sec5, and t-SNARE Sso1. The *sso1* mutant also showed abnormal BIC formation, having two focal points of symplastic effector accumulation, and in *exo70* and *sec5* mutants, intense Pwl2:mRFP signals remained inside the hyphae [10]. These phenotypes differ from the abnormal localization of effectors shown in the *Δrbf1*-invaded rice cells. The disruption of *RBF1* resulted in the dispersed accumulation of BIC marker effectors scattered around the unusual short primary hypha and the first bulbous IH (Figs 6–8). Therefore, Rbf1 probably does not act on the effector secretion process or machinery inside the fungal cell, although Rbf1 could involve the predominant localization of Exo70, Sec5, and Sso1 to BIC-associated cells by means of forming the focal BIC base.

*RBF1* putatively encodes 658 amino acids rich in glycine and alanine residues with short repetitive sequences (S1 Fig). A conserved domain search identified the region in Rbf1 (Ala^234^-Asp^360^) that shows a low similarity to a sequence conserved in DNA polymerase III gamma and tau subunits (accession PRK07764 in the NCBI conserved domain database [39]; E-value, 4.21 × 10^−3^). A deletion in this region (Rbf1Δ20) resulted in the dysfunction of Rbf1, suggesting the importance of the region for Rbf1 functioning. Although it is still unknown how these Rbf1 structural features are required for the focal accumulation of effector proteins or for the formation of the focal BIC base, it might be possible that Rbf1 participates in virulence as a chaperone to facilitate the translocation of symplastic effectors. Further studies on the functional domain in Rbf1 and its interacting factors are needed to reveal the mode of Rbf1 action on the focal BIC formation.

### The importance of focal BIC formation

The formation of the normal focal BIC structure was correlated to the pathogenicity of *M*. *oryzae* (Fig 7). Mutants lacking *RBF1* still showed dispersed BICs in the immune-depressed rice plants (Fig 8). Given that Rbf1 is involved in virulence exclusively via BIC formation, our data indicate that the focal BIC is crucial for the suppression of host immune responses to establish the biotrophic invasion (Fig 10).

What is the significance of the focal BIC structure? The dispersed BIC led to the enhanced induction of host immune responses and caused a severe defect in virulence (Figs 3 and 4). The translocation of a symplastic effector into rice cells was not abolished even in the dispersed BIC situation, but the data suggest that the amount of the translocated effector was significantly reduced (Fig 9). An impaired induction of the fungal cell lysis during the incompatible interaction also implies that the dispersed BIC caused a reduction in the translocation of an avirulence effector into host cells (S17 Fig). Based on these data, we hypothesize that the formation of the focal BIC structure is required for the translocation of sufficient amounts of symplastic effectors to evade host immunity.

Mutants lacking *RBF1* showed the short primary IH phenotype (Fig 6A and 6D). It is possible that the early differentiation of the filamentous primary hypha into the bulbous IH is also a result of the defect in the focal BIC formation at the tip of the primary hypha. Although further studies are needed to reveal the significance of the morphological switch of IH, our data imply that the focal BIC formation at the tip of the primary IH is deeply involved in the switch.

### Conclusion

We identified a novel virulence gene, *RBF1*, in *M*. *oryzae* and showed that Rbf1 is required for the focal BIC formation. The experimental evidence presented here indicate that the appropriate BIC formation is achieved by a fungal gene and the BIC structure is critical in establishing a biotrophic invasion by preventing the activation of host immune mechanisms (Fig 10), probably through the sufficient delivery of effectors into host cells. Studies of the molecular mechanism of Rbf1 function and the mode of the BIC action would be clues to elucidate the unique infection strategy developed in *M*. *oryzae*.

## Materials and Methods

### Fungal strains and transformations

*M*. *oryzae* strain ‘Ina86-137’ (race 007.0) was obtained from the NARO Gene Bank in Tsukuba, Japan (stock number MAFF101511). *Pyricularia* species used for genomic DNA-blot hybridization (S2 Fig) were also provided by the NARO Gene Bank. ‘Guy11’ was provided by Dr. Marie Nishimura of the NARO, Tsukuba, Japan in order to isolate *BAS4*. *Agrobacterium*-mediated transformation including the generation of *RBF1*-disrupted mutants was performed according to Saitoh *et al*. [25]. At least three transformants were selected for each vector construct based on fluorescence intensity, growth, conidiation on media plates, and virulence. Transformants used in this study are listed in S2 Table. Plasmid vectors to generate each transformant are listed in S3 Table with primer sequences used for PCR-amplification.

### Plant materials and growth conditions

Rice plants (*Oryza sativa* L. japonica) carrying the blast-resistance gene *Pia* and *Pish* [cv. Nipponbare (*Pia*)] was used unless otherwise stated. Transgenic rice lines expressing *GFP* under the CaMV *35S* promoter were generated using ‘Nipponbare Kanto-BL2’ harboring *Pii* and *Pish*. Rice seeds of ‘Nipponbare Kanto-BL2’ and ‘Nipponbare Kanto-BL5’ harboring *Pik* and *Pish* were kindly supplied by Dr. Hiroyuki Satoh of the NARO. Transgenic rice lines expressing *NahG* that had the ‘Nipponbare (*Pia*)’ background and were confirmed to contain a lowered SA level, were kindly provided by Dr. Chang-Jie Jiang of the NARO. Transgenic rice lines with the GFP-labeled PM were generated using ‘Nipponbare Kanto-BL2’ and pBIB-35S-EGFP-LTI6b, provided by Dr. S. Kurup of University of Cambridge. Cultivars Nipponbare (*Pia*) and Nipponbare Kanto-BL2 are compatible and Nipponbare Kanto-BL5 is incompatible to *M*. *oryzae* strain ‘Ina86-137’. Rice plants were hydroponically cultured in a chamber under a 14-h-light at 28°C and 10-h-dark at 25°C cycle as described in Tanabe *et al*. [40].

### Inoculation assays

The blast fungus was grown on oatmeal agar plates (30 g oatmeal, 5 g sugar, and 16 g agar l^−1^ water) for 7 days at 26°C in darkness, and then conidial formation was induced under a fluorescent light for 4 days. The crude conidial suspension was filtered through three layers of Miracloth (Calbiochem, La Jolla, CA, USA) to remove cell debris, washed with water, and collected by centrifugation as described in Tanabe *et al*. [40]. The washed conidial suspension was diluted with water to 2 × 10^5^ conidia ml^−1^ for spray-inoculation, 3 × 10^5^ conidia ml^−1^ for spot-inoculation, and 0.8 × 10^5^ conidia ml^−1^ for leaf sheath inoculation. Spray-inoculation assays were performed according to Chujo *et al*. [41] using 6-leaf-stage intact rice plants. For spot-inoculation assays, the 6^th^ leaf blades were detached from rice plants at the 6.5-leaf stage and placed on moistened filter paper in petri dishes. The leaf surfaces were stroked with absorbent cotton. Then, 5 μl of the washed conidial suspension was spotted on the leaf blades, followed by incubation at 25°C under 14-h-light and 10-h-dark cycles. For the leaf sheath assays, leaf sheaths of the 5^th^ or 6^th^ leaves were excised from rice plants at the 5.5- or 6.5-leaf stage and inoculated with the washed conidial suspension in the hollow interior of the detached leaf sheaths. For the preparation of dead leaf tissues, the excised sheaths (Fig 1C) or leaf blades (Fig 1D) were treated with 70% ethanol for 2 h and 100% ethanol overnight at 25°C, and then rehydrated with distilled water. The inoculated leaf sheaths were incubated at 25°C under darkness for 24–48 h. After incubation, the inner epidermal layers were observed using fluorescence microscopy. For the evaluation of IH growth (Fig 3C), the inoculated sheaths were fixed with a FAA solution [45% (v/v) ethanol, 5% (v/v) acetic acid, and 1.85% (v/v) formaldehyde] and degrees of hyphal growth were assessed for each appressorium under a microscope as described in Tanabe *et al*. [40]. For the observation of the cytoplasmic localization of effectors (Fig 2B), the infected leaf sheaths were plasmolyzed using sucrose as described in Khang *et al*. [14].

Blast disease development was quantified by quantitative genomic PCR analysis as described in Zellerhoff *et al*. [42]: the measurement of *M*. *oryzae 28S rDNA* relative to the rice *eEF-1α* gene. The primer sequences used are listed in S4 Table.

### qRT-PCR analysis

For the gene expression analysis in leaf blades, total RNA was isolated from two 1-cm long leaf sections per plant spotted with a conidial suspension. For the analysis in leaf sheaths, total RNA was isolated from two 1.5-cm long sections of inoculated leaf sheaths per plant. Total RNA was extracted using Sepasol RNA I Super (Nacalai Tesque, Kyoto, Japan). First strand cDNA was synthesized using the PrimeScript RT reagent kit (Takara Bio, Kusatsu, Japan). qRT-PCR was performed using SYBR Premix Ex Taq II (Takara Bio), and the relative levels of gene expression were quantified using MX3000P (Agilent Technologies Inc., Santa Clara, CA, USA). Data were normalized to the expression levels of *eEF-1α* in rice and *ACT1* in *M*. *oryzae*. Primer sequences are listed in S4 Table.

### Microscopy

Stereomicroscopy was performed using an MZ16F microscope (Leica, Wetzlar, Germany) and the images were obtained using a DP-70 camera (Olympus, Tokyo, Japan) (Fig 5). Light and fluorescence microscopy was performed using an Optiphoto (Nikon, Tokyo, Japan), and the images were obtained using a DP-71 camera (Olympus) (Fig 3E). Laser scanning confocal microscopy was performed using a TCS SP5 instrument (Leica) (Figs 1C, 2, 3C, 3D, 6A–6C, 7C–7E and 9A and S3 and S12–S15 Figs). Fluorescence was excited with an argon laser at 488 nm (GFP) or a green diode laser at 561 nm (mCherry) and detected at wavelengths of 500–520 nm for GFP or 600–620 nm for mCherry. In Figs 6D and 8, images were obtained using an epifluorescence microscope (DM6000B; Leica) equipped with a confocal laser scanning unit (CSU-X1; Yokogawa Electric, Tokyo, Japan), the laser units (Sapphire 488 and 561 nm; Coherent, Santa Clara, CA), dichroic mirror (DM-405/488/561), and emission filters (GFP, EM-520/35; mCherry, EM617/73). Fluorescence images were acquired using an EM-CCD camera (iXon897; Andor Technology PLC., Belfast, Northern Ireland, U.K.) with a 63× glycerol immersion objective (Leica). Images were processed and arranged using LAS AF software (Leica) and MetaMorph software (Molecular devices LLC, Sunnyvale, CA).

Time-lapse fluorescence imaging was performed according to the method of Mochizuki *et al*. [13]. Briefly, hand-sliced leaf sheath epidermal tissues were placed on agarose set on a glass slide and inoculated with a conidial suspension (5 × 10^5^ conidia ml^−1^). Then, the inoculated tissues were incubated at 25°C in the dark in a moist chamber for 12 h. After confirming appressorial penetration, the tissues were covered with dimethylpolysiloxane (200 cSt; Thermo Fisher Scientific Inc., Waltham, MA, USA), and a coverslip. GFP and mCherry fluorescence was observed using the confocal laser scanning system (CSU-X1) installed in the room at 25°C. Fluorescence images were acquired at 20-min intervals using an EM-CCD camera (iXon897; Andor Technology Plc., Belfast, UK) with a 20× long working distance objective (Leica).

### β-Glucuronidase (GUS) staining

To visualize hyphae, leaf blades inoculated with *GUS*-expressing transformants were incubated in GUS staining buffer [20 mM potassium phosphate buffer (pH 7.0), 0.1% TritonX-100 (v/v)] containing 1 mg ml^–1^ 5-bromo-4-chloro-3-indolyl-β-D-glucuronide (Nacalai Tesque) at 37°C until sufficient staining was observed.

### RNA-Seq analysis

Total RNA was extracted from WT- and KO-inoculated leaves, as well as water-spotted control leaves, using an RNeasy Mini Kit (Qiagen). PolyA-RNA was isolated using Dynal magnetic beads (Thermo Fisher Scientific Inc.). Double-stranded cDNA molecules were generated using random hexadeoxynucleotide primers and then sequenced using the Illumina RNA-Seq paired-end protocol on a HiSeq2000 (San Diego, CA, USA) with 90 cycles. Low quality bases and adapter sequences were trimmed using Trimmomatic v0.32 with the following parameter: ILLUMINACLIP:TruSeq3-PE.fa:2:30:10 LEADING:20 TRAILING:20 SLIDINGWINDOW:4:20 MINLEN:50 according to Bolger *et al*. [43]. Reads derived from ribosomal RNA, chloroplast and mitochondrial DNA of rice were removed by alignment to the reference sequences for those molecules using Bowtie v2.2.2 and TopHat v2.0.11 with the default parameters [44]. Furthermore, reads derived from the fungal transcripts were filtered out by alignment to the *M*. *oryzae* reference genome sequences (MG8). The preprocessed reads were aligned to the *O*. *sativa* ssp. japonica cv. Nipponbare reference genome sequence (IRGSP-1.0), containing the reference gene annotations obtained from RAP-DB and MSU Rice Genome Annotation databases, using Bowtie and TopHat [45,46]. Expression levels (FPKM values) for each locus were calculated and quartile normalization was applied using Cufflinks [47].

To select genes that were upregulated in KO-inoculated leaves, we first extracted genes with two-times more FPMK in KO-inoculated leaves than in WT-inoculated leaves, and then selected genes matching the following criteria: > 150 FPMK in KO-inoculated leaves and < 50 FPMK in mock-inoculated leaves, using Subio Platform ver. 1.1.7 software (Subio, Tokyo, Japan). The selected genes are listed in S1 Table.

### Quantification of PAs

Leaf blades were cut 5 mm away from the inoculated spots, and then, two 1-cm leaf sections per tube were extracted with 79% (v/v) ethanol containing 14% (v/v) water, 7% (v/v) acetonitrile, and 0.1% (v/v) acetic acid at 4°C for 24 h. The extracts were analyzed for the simultaneous determination of momilactones, phytocassanes, and sakuranetin using a HPLC-MS/MS spectrometer with combinations of the precursor and product ions (*m/z* 317/299 for phytocassanes A, D, and E; *m/z* 335/317 for phytocassane B; *m/z* 319/301 for phytocassane C; *m/z* 315/271 for momilactone A; *m/z* 331/269 for momilactone B; and *m/z* 287/167 for sakuranetin) in the multiple-reaction monitoring mode [48].

### Translocation assay of Pwl2:mCherry:NLS

Leaf sheaths of the 5^th^ leaves at the 5.5-leaf stage were excised and inoculated with a washed conidial suspension of the WT line or KO lines (line #1 and #2) transformed with *PWL2p*::*PWL2*:*mCherry*:*NLS*. After 24 h incubation, rice cells with mCherry signals were assessed under a fluorescence microscope and classified into three mCherry signal patterns indicated in Fig 9B. In total, 1,231, 1,008, and 1,012 infected loci were counted for the WT and two KO lines, respectively. The values were the average of four or five independent experiments using three leaf sheathes for each experiment.

### Data Availability

The nucleotide sequence data of *RBF1* will appear in the DDBJ/EMBL/GenBank database under the accession number LC146480.

## Supporting Information

S1 Table Genes upregulated in the rice leaves inoculated with an *RBF1*-knockout mutant. (XLSX)

S2 Table *Magnaporthe oryzae* transformants used in this study. (XLSX)

S3 Table Oligonucleotide primers used for vector construction. (XLSX)

S4 Table Oligonucleotide primers used in quantitative analysis and others. (XLSX)

S1 Fig Comparison of amino acid sequences encoded by *RBF1* in different *Magnaporthe oryzae* strains. Rbf1 sequences deduced from the DNA sequences in different rice blast fungal strains (‘70–15’, ‘Y34’, and ‘P131’) found in the database are aligned with the Rbf1 of ‘Ina86-137’ (accession number LC146480) and its unexpectedly generated dysfunctional mutant Rbf1Δ20. The arrow indicates the secretion signal sequence. The broken-line arrow indicates the region identified to be similar with a model domain in the DNA polymerase III gamma and tau subunits (accession in the NCBI’s conserved domain database, PRK07764; E-value, 4.21 × 10^−3^). The double lines indicate a glycine-rich repetitive sequence.
(PDF)

S2 Fig Distribution of *RBF1* homologs analyzed by genomic DNA-blot hybridization. Genomic DNA was extracted from the blast fungus strains isolated from the different gramineous plants listed in **(A)** and digested with *Hin*dIII and *Eco*RI. DNA blots were hybridized with a mixture of three probes corresponding to the *RBF1* open reading frame shown in **(B)**: (1) 64–232, (2) 573–870, and (3) 1,539–1,995 (numbers indicate the nucleotide position from the start codon). The estimated size of the band detected from the ‘Ina86-137’ strain is 1.95 kb. As a result, positive bands were detected in *M*. *oryzae* rice isolates and their closely-related strains **(C)**. Osa, *Oryza sativa*; Pmi, *Panicum miliaceum*; Eaf, *Eleusine africana*; Hvu, *Hordeum vulgare*; Lmu, *Lolium multiflorum*; Zma, *Zea mays*; Asa, *Avena sativa*; Dci, *Digitaria ciliaris*; Ssp, *Sasa* sp.; and Pba, *Phyllostachys bambusoides*.
(PDF)

S3 Fig Signal sequence in Rbf1 functions as a secretion signal. **(A)** Accumulation of the wild-type Rbf1 in the BIC at the tip of the primary invasive hypha. **(B)** Hyphal accumulation of Rbf1 translated from the mutant *RBF1* that lacks the region encoding the secretion signal sequence. **(C)** BIC accumulation of mCherry translated from the *mCherry* fused with the signal sequence of *RBF1*. Rice leaf sheaths were inoculated with the WT-based transformants, and observed by confocal microscopy at 30–32 hpi. Images of mCherry signals were merged with differential interference contrast images. Bar = 10 μm.
(PDF)

S4 Fig Construction of *RBF1*-disrupted lines carrying *GFP* (Δ*rbf1-1*). **(A)** Scheme of *RBF1* disruption via *Agrobacterium*-mediated homologous recombination. The T-DNA region in the disruption vector pCAMBIA-*RBF1*-KO contains the 734-bp upper flanking region (UFR) of the start codon, a *GFP-HPT* cassette, and the 638-bp downstream flanking region (DFR) of the stop codon in *RBF1*. Homologous recombination occurring in the UFR and DFR results in the replacement of the *RBF1* open reading flame with the *GFP*-*HPT* cassette, thus the resulting knockout lines (*Δrbf1-1*) express GFP from the *RBF1* promoter and are hygromycin resistant. Open boxes and shaded boxes indicate the *att*B region of the Gateway cloning system and the T-DNA border region, respectively. E, *Eco*RI site; H, *Hin*dIII site. **(B)** Genomic structure of the transformant (*RBF1p*::*GFP*) in which the T-DNA region of pCAMBIA-*RBF1*-KO was inserted into the fungal genome ectopically. The ectopic transformant was used to monitor the *RBF1* expression by live cell imaging. **(C)** Genomic DNA-blot hybridization analysis of the wild-type ‘Ina86-137’ strain (WT), ectopic transformant (*RBF1p*::*GFP*), and two independent *RBF1*-disrupted mutants (*Δrbf1-1* line 1 and line 2).
(PDF)

S5 Fig *RBF1*-disruption mutant develops normally to appressoria *in vitro*. **(A)** Diameters of the colonies of wild-type (WT) and *RBF1*-disruption mutant (KO) formed on PDA medium after 10 days of culturing at 25°C. Data are represented as mean values ± SE for six colonies. **(B)** Number of spores collected from a colony formed on PDA medium after 10 days of culturing at 25°C. Data are represented as mean values ± SE for five colonies. **(C)** Morphology of germinated spores and appressoria from WT and KO on glass plates. Photos were taken 12 h after the preparation of a conidial suspension. Bar = 20 μm. **(D)** Rate of germination and appressoria formation in the WT and KO on glass plates after 18 h imbibition. Conidia, non-germinated conidia; germination, germinated conidia; appressoria formation, germinated conidia with an appressorium. Data are the average of two biological repeats. In total, 1,134 WT and 1,020 KO conidia were counted.
(PDF)

S6 Fig Total number of lesions formed in leaves is not affected by a lack of *RBF1*. Rice plants were sprayed with a conidial suspension of the wild-type (WT) strain, an *RBF1*-knockout line (KO), and a gene complementation line (KO*+RBF1*), and the number of lesions in the 7-cm sections of the 6^th^ leaves at 5 dpi was counted. Data are represented as the mean values ± SE (*n* = 5 plants). No significant difference was detected using Student’s *t*-test.
(PDF)

S7 Fig A lack of *RBF1* causes a drastic reduction in proliferation in rice leaves. **(A)** Defect in lesion formation in the *RBF1*-knockout line. Excised rice leaf blades were spotted with a conidial suspension of the wild-type (WT) strain, *Δrbf1-1* (KO), and two gene complementation lines (KO+*RBF1*), and incubated for 6 days. Bar = 5 mm. **(B)** Proliferation of *M*. *oryzae* in rice leaf blades at 6 dpi evaluated by a quantitative PCR method. Fungal genomic DNA was isolated from the spot-inoculated leaf blades and the amount of *M*. *oryzae* 28S rDNA (*Mo28S*) relative to rice *eEF-1α* (*OsEF1*) was determined. Data are represented as mean values ± SE (*n* = 5 plants).
(PDF)

S8 Fig Construction of *RBF1*-disrupted lines without *GFP* (Δ*rbf1-2*). **(A)** Scheme of the *RBF1* disruption via *Agrobacterium*-mediated homologous recombination. The T-DNA region in the disruption vector pCAMBIA-*RBF1*-KO2 contains the 734-bp upper flunking region (UFR) of the start codon, a *TrpCp*::*HPT* cassette, and the 638-bp downstream flunking region (DFR) of the stop codon in *RBF1*. Homologous recombination occurring in the UFR and DFR results in the replacement of the *RBF1* open reading flame with the *HPT* cassette, thus the resulting knockout lines (*Δrbf1-2*) are hygromycin resistant. Open boxes and shaded boxes indicate the *att*B region of the Gateway cloning system and the T-DNA border region, respectively. E, *Eco*RI site; H, *Hin*dIII site. **(B)** Genomic structure of the transformant in which the T-DNA region of pCAMBIA-*RBF1*-KO2 was inserted into the fungal genome ectopically. Positions of primers used in **(C)** are indicated. **(C)** Genomic PCR analysis of the wild-type ‘Ina86-137’ strain (WT), two independent *RBF1*-disrupted mutants (*Δrbf1-2* line 1 and line 2), and an ectopic transformant. **(D)** Defect in lesion formation in the *RBF1*-knockout lines (*Δrbf1-1* and *Δrbf1-2*). Bar = 5 mm.
(PDF)

S9 Fig Symptoms on the rice leaf blades spot-inoculated with the WT- or KO-based transformant and the GUS staining images. The broken lines indicate the hand-sectioned sites shown in Fig 3E.
(PDF)

S10 Fig *RBF1* does not affect the infection-induced production of a flavonoid phytoalexin, sakuranetin. **(A)** qRT-PCR analysis of the expression of *NOMT* (Os12g0240900), which encodes the key enzyme for sakuranetin biosynthesis, in the inoculated rice leaf blades at 2 dpi. Data are represented as the mean values ± SE of four individual leaves. **(B)** Quantification of sakuranetin in inoculated leaf blades. Data of five to seven independent extracts in two inoculation assays are represented as mean values ± SE. No significant differences between WT and *Δrbf1-1* (KO) were detected using Student’s *t*-test. Sakuranetin was not detected in the mock-inoculated leaves (n. d.).
(PDF)

S11 Fig Activation of defense-related genes by *Magnaporthe oryzae* infection is impaired in *NahG*-expressing and ABA-treated rice. Rice leaf blades were spot-inoculated with a conidial suspension of the WT strain, and total RNA was extracted at 2 dpi for qRT-PCR analysis. Data are represented as the mean values ± SE (*n* = 4 plants). The expression of *OsWRKY45* (Os05g0322900) and *SalT* (Os01g0348900) was also examined as indicators for SA and ABA signaling, respectively.
(PDF)

S12 Fig Comparison of Bas4:mCherry localization and EIHM between WT and KO. **(A)** Confocal images of rice leaf sheath cells infected by the WT or *Δrbf1-1* (KO) line harboring *BAS4p*::*BAS4*:*mCherry* at 36 hpi. Arrow indicates the focal accumulation of the effector at the predicted BIC position. **(B)** Confocal images of rice leaf sheath cells expressing GFP:LTI6B at 30 hpi with the WT or *Δrbf1-2* (KO) line harboring *BAS4p*::*BAS4*:*mCherry*. Arrow indicates the aggregation of EIHM at the BIC position. Asterisks, appressoria. Bar = 10 μm.
(PDF)

S13 Fig Dispersed localization of an effector protein in Δ*rbf1*-invaded rice cells. **(A)** Quantitative RT-PCR analysis of the expression of an effector candidate gene in *M*. *oryzae* (*MGG_10010*) in conidia and inoculated rice leaf blades. The vertical axis indicates the amount of transcripts relative to that from the *M*. *oryzae* actin gene (*MoACT1*). Data are represented as mean values ± standard error (SE) (*n* = 3 plants). **(B)** Confocal images of rice leaf sheath cells infected by the WT or *Δrbf1-1* (KO) line harboring *010p*::*010*:*mCherr*y, which encodes an mCherry fusion of MGG_10010 at 36 hpi. Asterisks, appressoria. Bar = 10 μm.
(PDF)

S14 Fig Confocal images of rice leaf sheath cells infected by the WT or Δ*rbf1-2* (KO) line harboring *PWL2p*::*PWL2*:*GFP&BAS4p*::*BAS4*:*mCherry* at 36 hpi. Asterisks, appressoria. Bar = 10 μm.
(PDF)

S15 Fig Comparison of BIC-associated accumulation of host cytosol between WT and KO. Leaf sheaths of transgenic rice with *35S*::*GFP* were inoculated with the WT or *Δrbf1-2* (KO) line transformed with *PWL2p*::*PWL2*:*mCherry* **(A)** or *BAS4p*::*BAS4*:*mCherry* **(B)**, and observed using a confocal microscope at 30 hpi. Arrows indicate the focal accumulation of effectors with rice cytosol at BICs. Bar = 10 μm.
(PDF)

S16 Fig qRT-PCR analysis of *mCherry* expression in transformants containing *PWL2p*::*PWL2*:*mCherry*:*NLS*. Expression levels among three transformant lines, one having the WT background and the others having the *RBF1*-knockout background, were confirmed to be similar at 24 hpi in leaf sheaths. *n* = 13–15 plants.
(PDF)

S17 Fig Comparison of the incompatible interactions between WT and KO. **(A)** Ratio of the sites showing invasive-hyphal lysis to the total infection sites. Rice leaf sheaths of a resistant cultivar were inoculated with the WT or *Δrbf1-1* (KO) line harboring *TEFp*::*mCherry*, and the number of infection sites showing the mCherry leakage was counted under a fluorescence microscope at 30 hpi. Data are represented as the mean percentages ± SE (*n* = 5 plants). Student’s *t*-test was performed on arcsine-transformed data between WT and KO (*, *P* < 0.05). **(B)** Images of the 5^th^ leaf blades of the resistant cultivar at 4 days post spray-inoculation. Bar = 5 mm.
(PDF)

S1 Movie Dynamics of *RBF1* expression during the early infection stages captured by a time-lapse fluorescence imaging method. Inner epidermis of rice leaf sheath was inoculated with *Magnaporthe oryzae* transformed with *RBF1p*::*GFP*, and GFP fluorescence was acquired at 20-min intervals from 18 hpi for 26 h. *Z*-series of confocal fluorescence images for GFP (indicating *RBF1* expression) was stacked. Bar = 20 μm.
(MOV)

S2 Movie Dynamics of *PWL2* expression during the early infection stages captured by a time-lapse fluorescence imaging method. Inner epidermis of rice leaf sheath was inoculated with *Magnaporthe oryzae* transformed with *PWL2p*::*GFP*, and GFP fluorescence was acquired at 30-min intervals from 18 hpi. This movie comprises 53 images acquired from 21 hpi for 26 h. *Z*-series of confocal fluorescence images for GFP (indicating *PWL2* expression) was stacked. Bar = 20 μm.
(MOV)
