# Supplementary material for: Magnaporthe oryzae Glycine-Rich Secretion Protein, Rbf1 Critically Participates in Pathogenicity through the Focal Formation of the Biotrophic Interfacial Complex
Source: PLoS Pathog. 2016 Oct 6;12(10):e1005921. doi: 10.1371/journal.ppat.1005921 (PMC5053420; doi:10.1371/journal.ppat.1005921)
Supplement: S1 Fig — Rbf1 sequences deduced from the DNA sequences in different rice blast fungal strains (‘70–15’, ‘Y34’, and ‘P131’) found in the database are aligned with the Rbf1 of ‘Ina86-137’ (accession number LC146480) and its unexpectedly generated dysfunctional mutant Rbf1Δ20. The arrow indicates the secretion signal sequence. The broken-line arrow indicates the region identified to be similar with a model domain in the DNA polymerase III gamma and tau subunits (accession in the NCBI’s conserved domain database, PRK07764; E-value, 4.21 × 10^−3^). The double lines indicate a glycine-rich repetitive sequence. (PDF) [file ppat.1005921.s005.pdf]

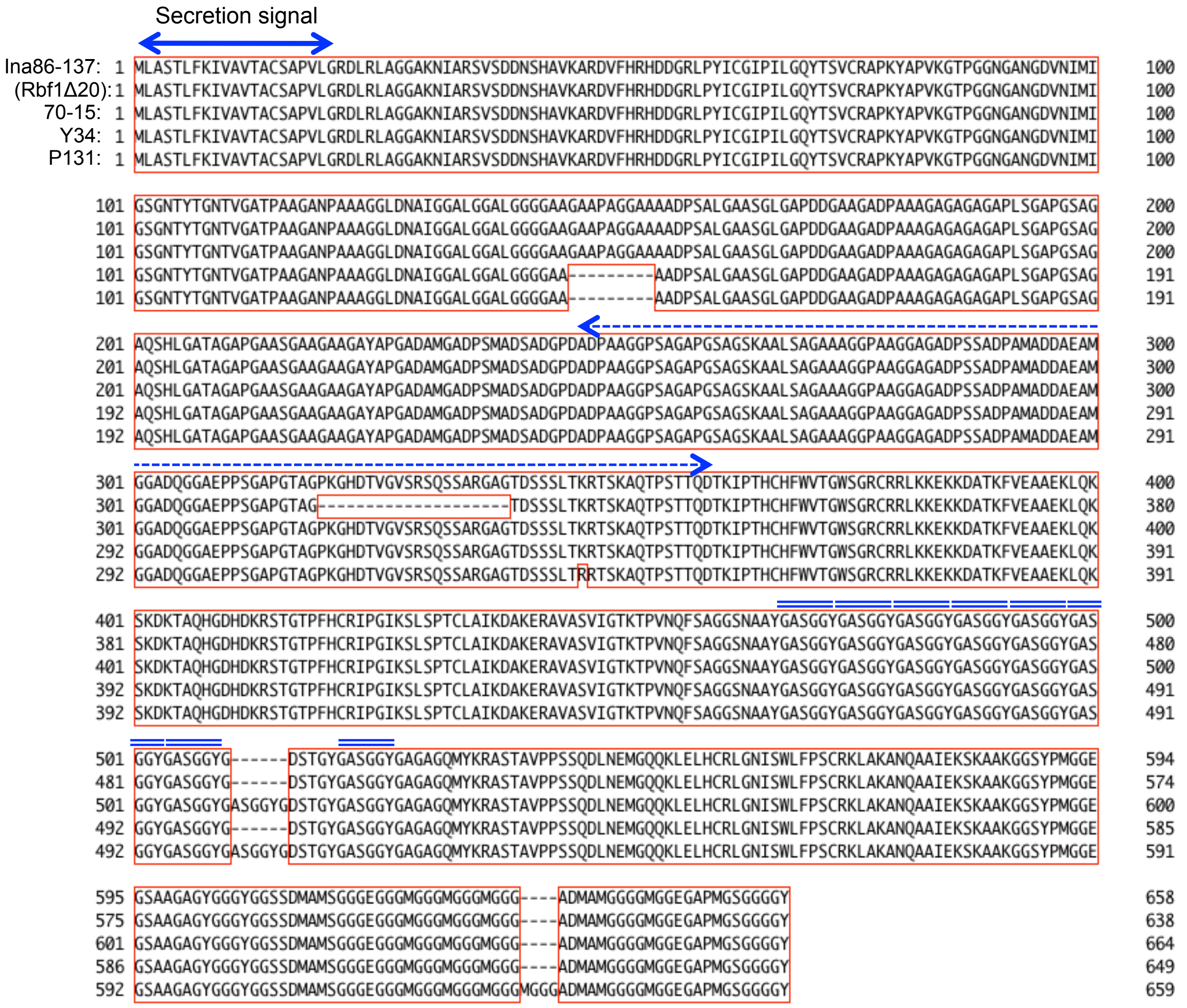

S1 Fig. Comparison of amino acid sequences encoded by *RBF1* in different *Magnaporthe oryzae* strains.
