# Supplementary material for: Magnaporthe oryzae Glycine-Rich Secretion Protein, Rbf1 Critically Participates in Pathogenicity through the Focal Formation of the Biotrophic Interfacial Complex
Source: PLoS Pathog. 2016 Oct 6;12(10):e1005921. doi: 10.1371/journal.ppat.1005921 (PMC5053420; doi:10.1371/journal.ppat.1005921)
Supplement: S2 Fig — Genomic DNA was extracted from the blast fungus strains isolated from the different gramineous plants listed in (A) and digested with HindIII and EcoRI. DNA blots were hybridized with a mixture of three probes corresponding to the RBF1 open reading frame shown in (B): (1) 64–232, (2) 573–870, and (3) 1,539–1,995 (numbers indicate the nucleotide position from the start codon). The estimated size of the band detected from the ‘Ina86-137’ strain is 1.95 kb. As a result, positive bands were detected in M. oryzae rice isolates and their closely-related strains (C). Osa, Oryza sativa; Pmi, Panicum miliaceum; Eaf, Eleusine africana; Hvu, Hordeum vulgare; Lmu, Lolium multiflorum; Zma, Zea mays; Asa, Avena sativa; Dci, Digitaria ciliaris; Ssp, Sasa sp.; and Pba, Phyllostachys bambusoides. (PDF) [file ppat.1005921.s006.pdf]

A

	Source	MAFF No.
Ina86-137	Rice	101511
ARC-P91-15B	Rice	101191
Kyu89-246	Rice	101506
TH68-126	Rice	101517
Ai79-142	Rice	101520
GFOS 4-1-1	Rice	101449
STPM 1-1-1	Proso millet	239339
SZPM 1-2-2	Proso millet	239342
NI 1006	Finger millet	239338
C-2-1	Barley	511494
IRBY 1-1	Italian ryegrass	511461
MBT 1-3	Maize	511464
AP4-1	Oat	306606
IBDS 1-1-1	Southern crabgrass	101027
IBDS 7-1-1	Southern crabgrass	239325
INA-B-92-45	Bamboo grass	240225
INA-B-83-19	Bamboo	240226

B

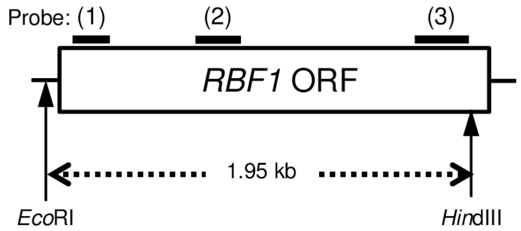

C

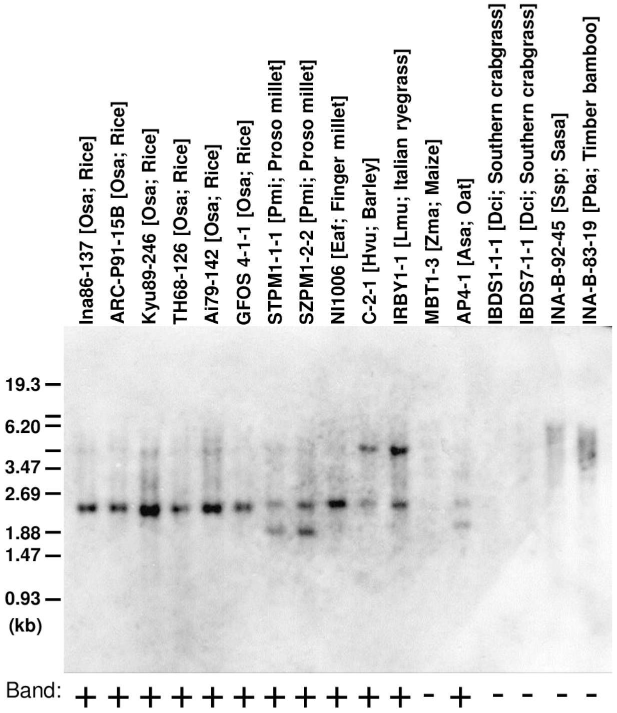

S2 Fig. Distribution of *RBF1* homologs analyzed by genomic DNA-blot hybridization.
