# Supplementary figures and images for: Magnaporthe oryzae Glycine-Rich Secretion Protein, Rbf1 Critically Participates in Pathogenicity through the Focal Formation of the Biotrophic Interfacial Complex
Source: PLoS Pathog. 2016 Oct 6;12(10):e1005921. doi: 10.1371/journal.ppat.1005921 (PMC5053420; doi:10.1371/journal.ppat.1005921)

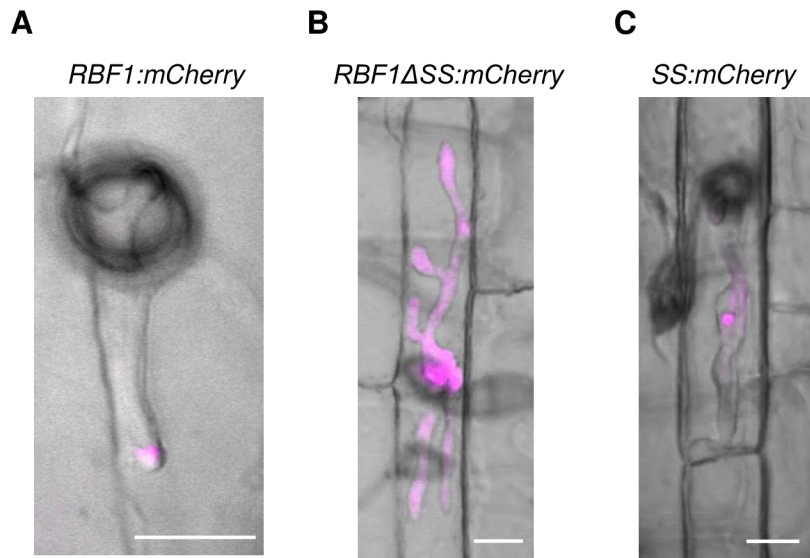

**S3 Fig. Signal sequence in Rbf1 functions as a secretion signal.**

Supplement: S3 Fig — (A) Accumulation of the wild-type Rbf1 in the BIC at the tip of the primary invasive hypha. (B) Hyphal accumulation of Rbf1 translated from the mutant RBF1 that lacks the region encoding the secretion signal sequence. (C) BIC accumulation of mCherry translated from the mCherry fused with the signal sequence of RBF1. Rice leaf sheaths were inoculated with the WT-based transformants, and observed by confocal microscopy at 30–32 hpi. Images of mCherry signals were merged with differential interference contrast images. Bar = 10 μm. (PDF) [file ppat.1005921.s007.pdf]

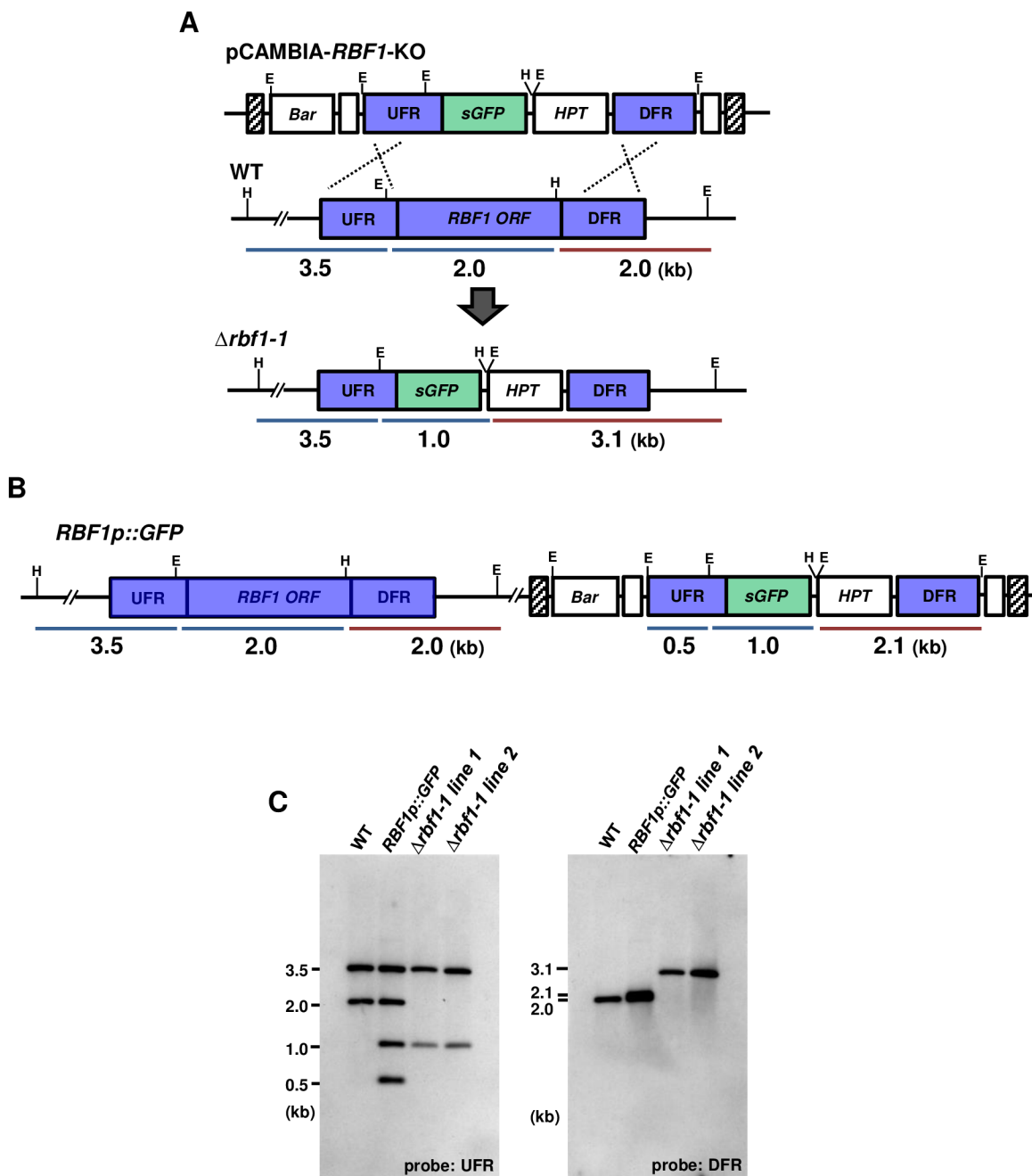

S4 Fig. Construction of *RBF1*-disrupted lines carrying *GFP* ( $\Delta rbf1-1$ ).

Supplement: S4 Fig — (A) Scheme of RBF1 disruption via Agrobacterium-mediated homologous recombination. The T-DNA region in the disruption vector pCAMBIA-RBF1-KO contains the 734-bp upper flanking region (UFR) of the start codon, a GFP-HPT cassette, and the 638-bp downstream flanking region (DFR) of the stop codon in RBF1. Homologous recombination occurring in the UFR and DFR results in the replacement of the RBF1 open reading flame with the GFP-HPT cassette, thus the resulting knockout lines (Δrbf1-1) express GFP from the RBF1 promoter and are hygromycin resistant. Open boxes and shaded boxes indicate the attB region of the Gateway cloning system and the T-DNA border region, respectively. E, EcoRI site; H, HindIII site. (B) Genomic structure of the transformant (RBF1p::GFP) in which the T-DNA region of pCAMBIA-RBF1-KO was inserted into the fungal genome ectopically. The ectopic transformant was used to monitor the RBF1 expression by live cell imaging. (C) Genomic DNA-blot hybridization analysis of the wild-type ‘Ina86-137’ strain (WT), ectopic transformant (RBF1p::GFP), and two independent RBF1-disrupted mutants (Δrbf1-1 line 1 and line 2). (PDF) [file ppat.1005921.s008.pdf]

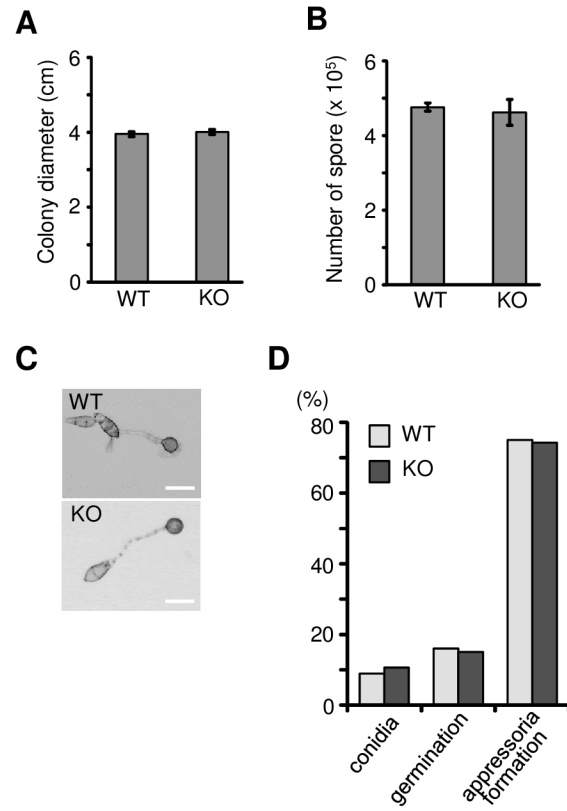

**S5 Fig. *RBF1*-disruption mutant develops normally to appressoria *in vitro*.**

Supplement: S5 Fig — (A) Diameters of the colonies of wild-type (WT) and RBF1-disruption mutant (KO) formed on PDA medium after 10 days of culturing at 25°C. Data are represented as mean values ± SE for six colonies. (B) Number of spores collected from a colony formed on PDA medium after 10 days of culturing at 25°C. Data are represented as mean values ± SE for five colonies. (C) Morphology of germinated spores and appressoria from WT and KO on glass plates. Photos were taken 12 h after the preparation of a conidial suspension. Bar = 20 μm. (D) Rate of germination and appressoria formation in the WT and KO on glass plates after 18 h imbibition. Conidia, non-germinated conidia; germination, germinated conidia; appressoria formation, germinated conidia with an appressorium. Data are the average of two biological repeats. In total, 1,134 WT and 1,020 KO conidia were counted. (PDF) [file ppat.1005921.s009.pdf]

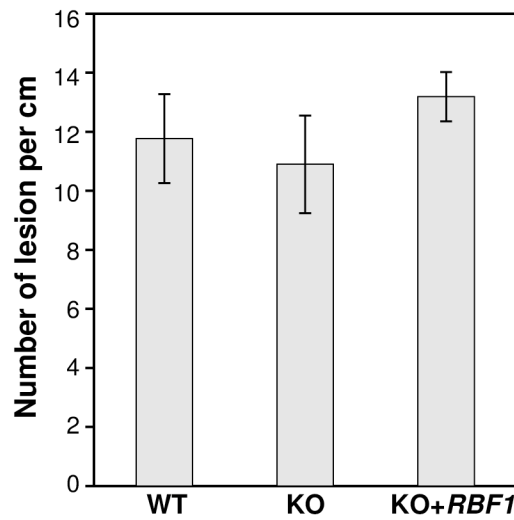

**S6. Total number of lesions formed in leaves is not affected by a lack of *RBF1*.**

Supplement: S6 Fig — Rice plants were sprayed with a conidial suspension of the wild-type (WT) strain, an RBF1-knockout line (KO), and a gene complementation line (KO+RBF1), and the number of lesions in the 7-cm sections of the 6th leaves at 5 dpi was counted. Data are represented as the mean values ± SE (n = 5 plants). No significant difference was detected using Student’s t-test. (PDF) [file ppat.1005921.s010.pdf]

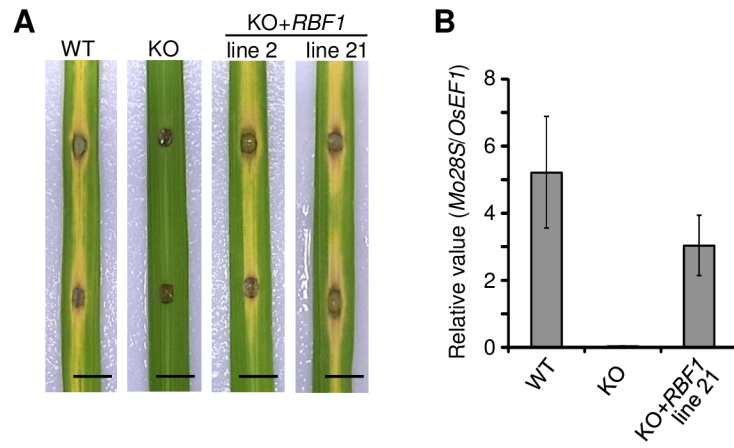

**S7 Fig. A lack of *RBF1* causes a drastic reduction in proliferation in rice leaves.**

Supplement: S7 Fig — (A) Defect in lesion formation in the RBF1-knockout line. Excised rice leaf blades were spotted with a conidial suspension of the wild-type (WT) strain, Δrbf1-1 (KO), and two gene complementation lines (KO+RBF1), and incubated for 6 days. Bar = 5 mm. (B) Proliferation of M. oryzae in rice leaf blades at 6 dpi evaluated by a quantitative PCR method. Fungal genomic DNA was isolated from the spot-inoculated leaf blades and the amount of M. oryzae 28S rDNA (Mo28S) relative to rice eEF-1α (OsEF1) was determined. Data are represented as mean values ± SE (n = 5 plants). (PDF) [file ppat.1005921.s011.pdf]

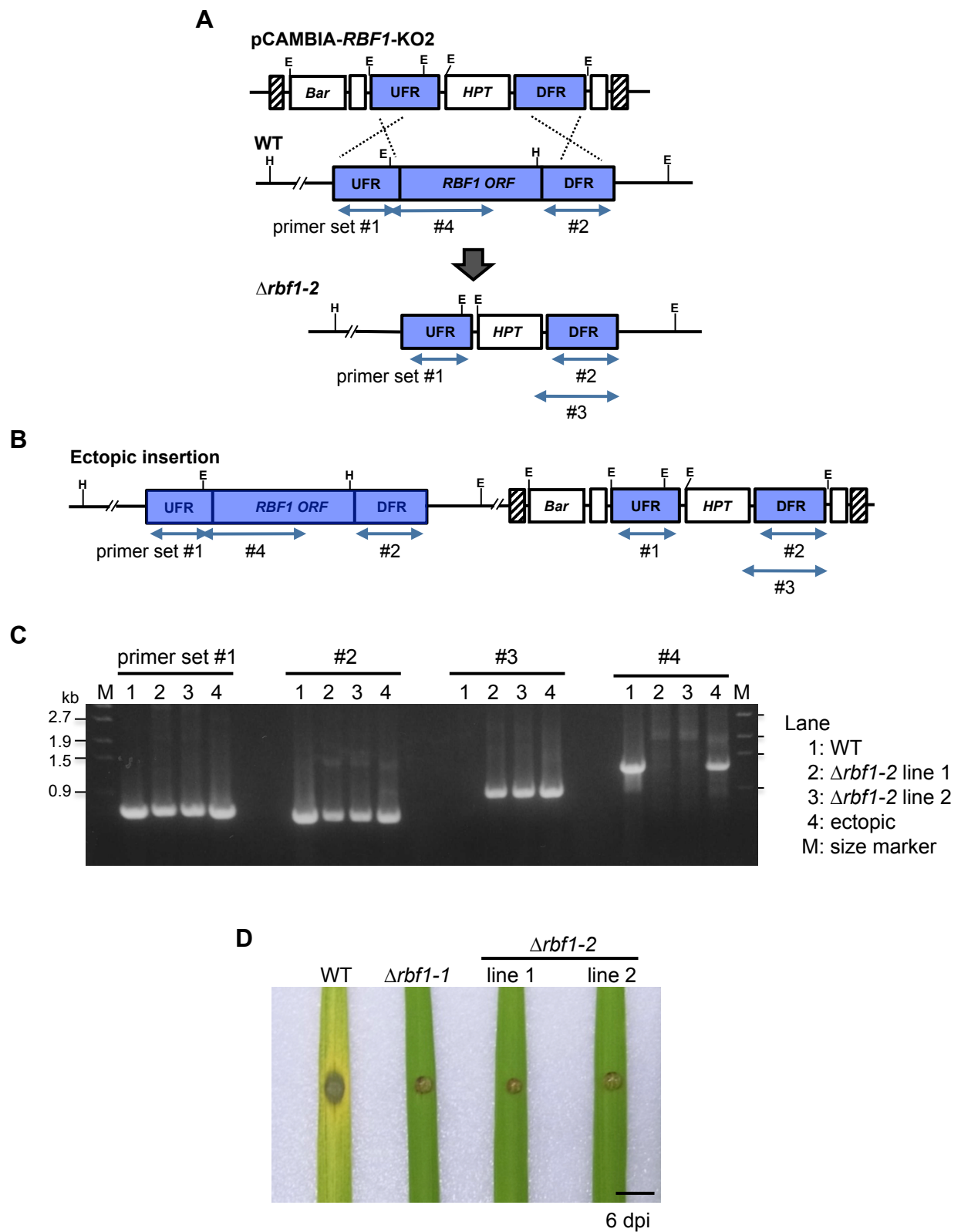

**S8 Fig. Construction of *RBF1*-disrupted lines without *GFP* (Δ*rbf1-2*).**

Supplement: S8 Fig — (A) Scheme of the RBF1 disruption via Agrobacterium-mediated homologous recombination. The T-DNA region in the disruption vector pCAMBIA-RBF1-KO2 contains the 734-bp upper flunking region (UFR) of the start codon, a TrpCp::HPT cassette, and the 638-bp downstream flunking region (DFR) of the stop codon in RBF1. Homologous recombination occurring in the UFR and DFR results in the replacement of the RBF1 open reading flame with the HPT cassette, thus the resulting knockout lines (Δrbf1-2) are hygromycin resistant. Open boxes and shaded boxes indicate the attB region of the Gateway cloning system and the T-DNA border region, respectively. E, EcoRI site; H, HindIII site. (B) Genomic structure of the transformant in which the T-DNA region of pCAMBIA-RBF1-KO2 was inserted into the fungal genome ectopically. Positions of primers used in (C) are indicated. (C) Genomic PCR analysis of the wild-type ‘Ina86-137’ strain (WT), two independent RBF1-disrupted mutants (Δrbf1-2 line 1 and line 2), and an ectopic transformant. (D) Defect in lesion formation in the RBF1-knockout lines (Δrbf1-1 and Δrbf1-2). Bar = 5 mm. (PDF) [file ppat.1005921.s012.pdf]

**A**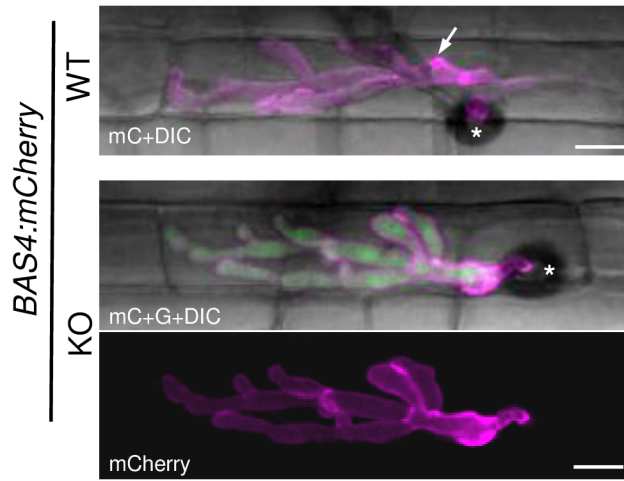**B**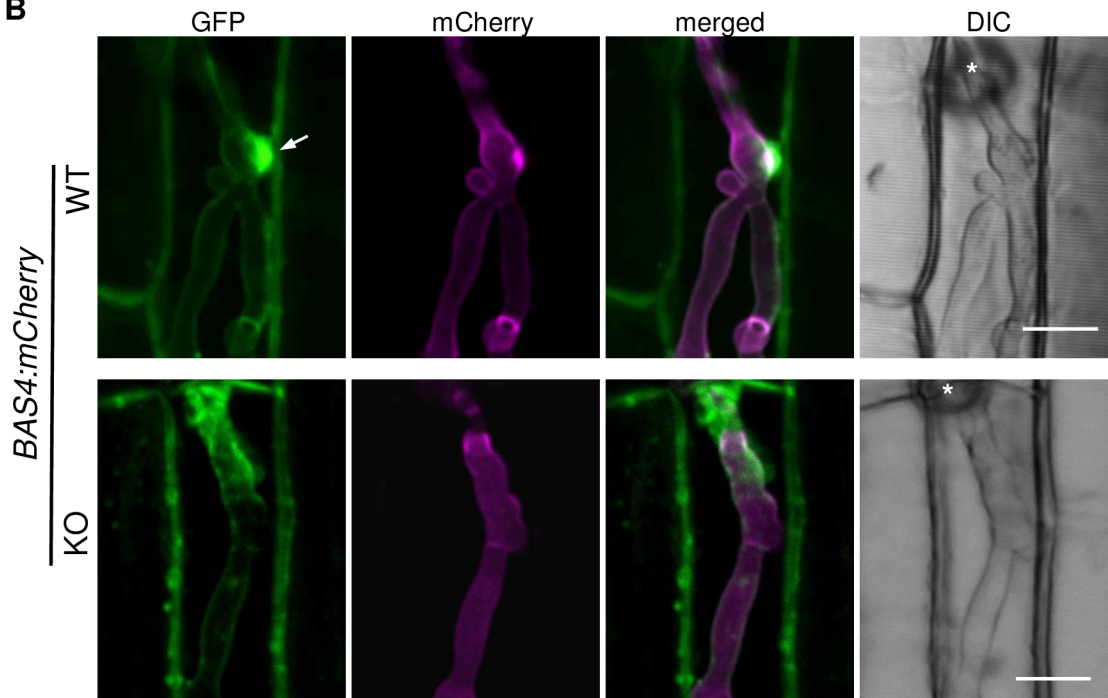

**S12 Fig. Comparison of Bas4:mCherry localization and EIHM between WT and KO.**

Supplement: S12 Fig — (A) Confocal images of rice leaf sheath cells infected by the WT or Δrbf1-1 (KO) line harboring BAS4p::BAS4:mCherry at 36 hpi. Arrow indicates the focal accumulation of the effector at the predicted BIC position. (B) Confocal images of rice leaf sheath cells expressing GFP:LTI6B at 30 hpi with the WT or Δrbf1-2 (KO) line harboring BAS4p::BAS4:mCherry. Arrow indicates the aggregation of EIHM at the BIC position. Asterisks, appressoria. Bar = 10 μm. (PDF) [file ppat.1005921.s016.pdf]

**A**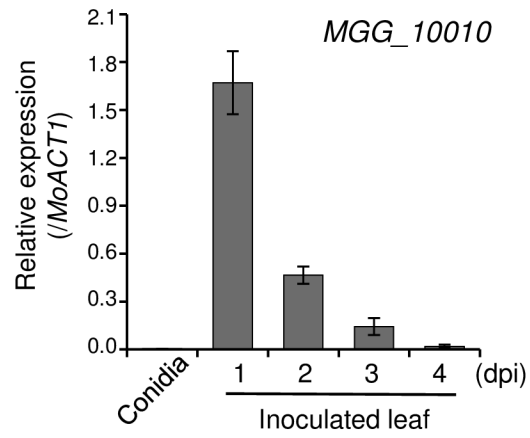**B**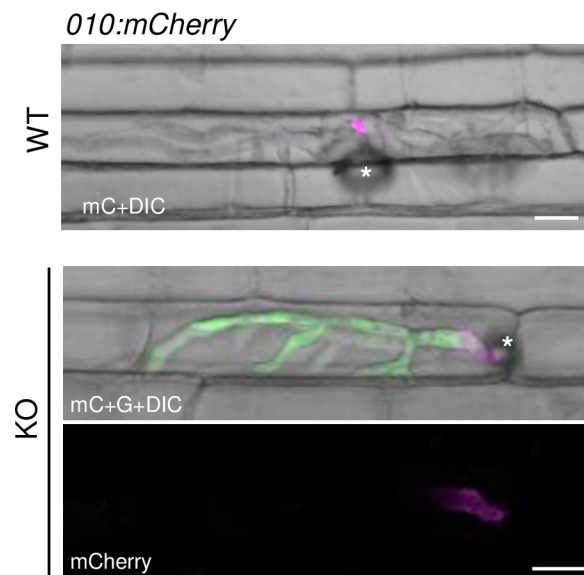

**S13 Fig. Dispersed localization of an effector protein in  $\Delta rbf1$ -invaded rice cells.**

Supplement: S13 Fig — (A) Quantitative RT-PCR analysis of the expression of an effector candidate gene in M. oryzae (MGG_10010) in conidia and inoculated rice leaf blades. The vertical axis indicates the amount of transcripts relative to that from the M. oryzae actin gene (MoACT1). Data are represented as mean values ± standard error (SE) (n = 3 plants). (B) Confocal images of rice leaf sheath cells infected by the WT or Δrbf1-1 (KO) line harboring 010p::010:mCherry, which encodes an mCherry fusion of MGG_10010 at 36 hpi. Asterisks, appressoria. Bar = 10 μm. (PDF) [file ppat.1005921.s017.pdf]

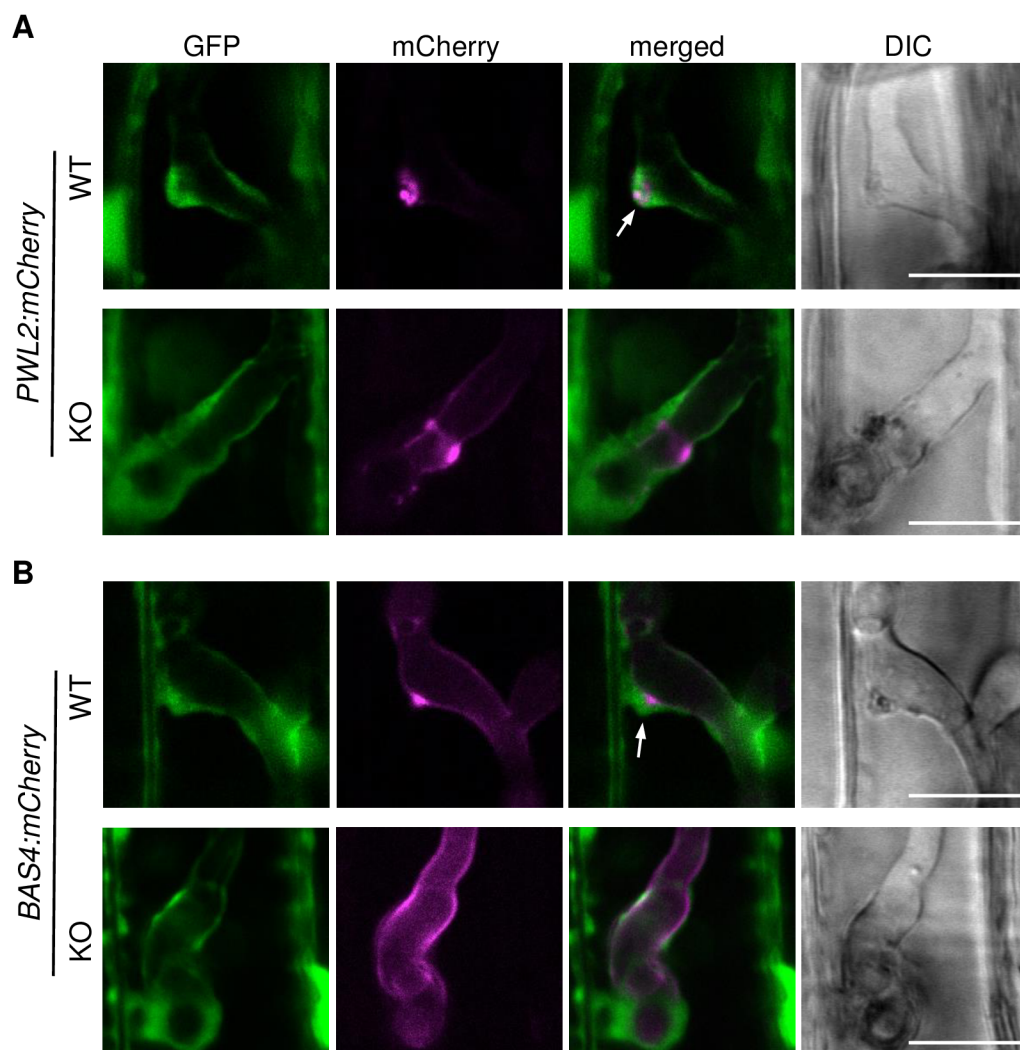

**S15 Fig. Comparison of BIC-associated accumulation of host cytosol between WT and KO.**

Supplement: S15 Fig — Leaf sheaths of transgenic rice with 35S::GFP were inoculated with the WT or Δrbf1-2 (KO) line transformed with PWL2p::PWL2:mCherry (A) or BAS4p::BAS4:mCherry (B), and observed using a confocal microscope at 30 hpi. Arrows indicate the focal accumulation of effectors with rice cytosol at BICs. Bar = 10 μm. (PDF) [file ppat.1005921.s019.pdf]

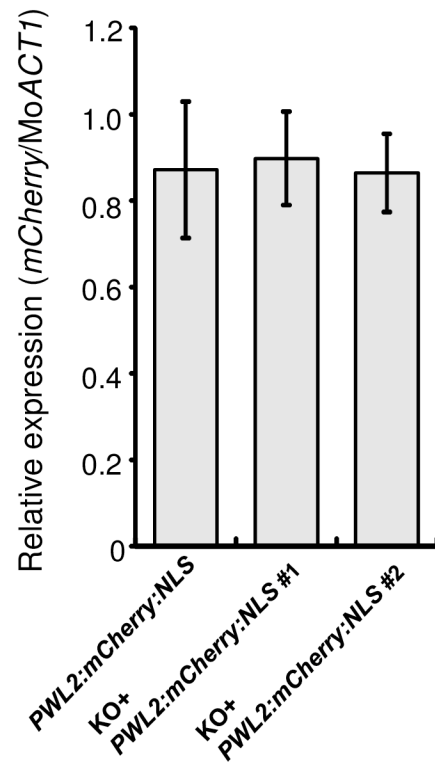

**S16 Fig.** qRT-PCR analysis of *mCherry* expression in transformants containing *PWL2p::PWL2:mCherry:NLS*.

Supplement: S16 Fig — Expression levels among three transformant lines, one having the WT background and the others having the RBF1-knockout background, were confirmed to be similar at 24 hpi in leaf sheaths. n = 13–15 plants. (PDF) [file ppat.1005921.s020.pdf]

**A**

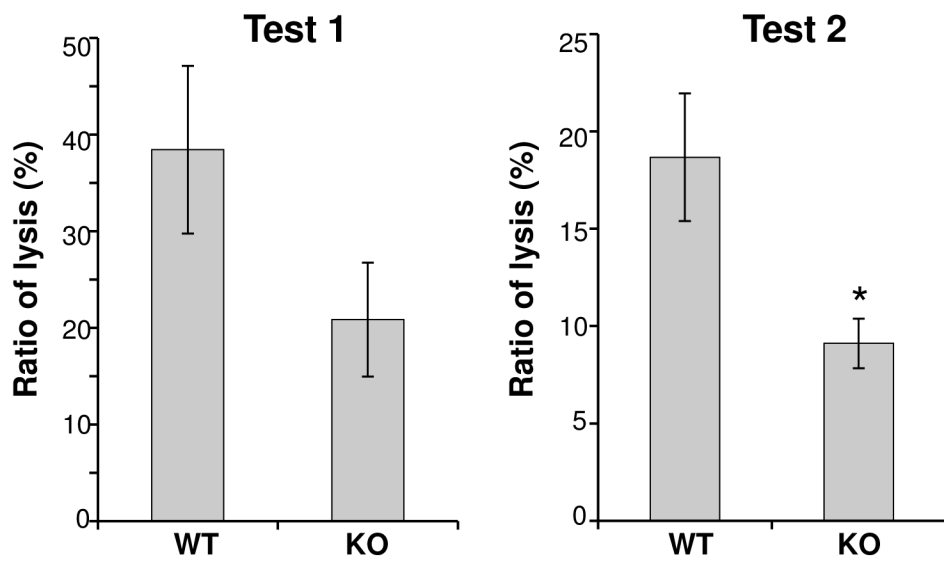

**B**

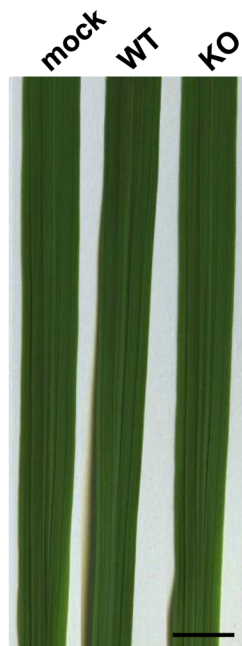

**S17 Fig. Comparison of the incompatible interactions between WT and KO.**

Supplement: S17 Fig — (A) Ratio of the sites showing invasive-hyphal lysis to the total infection sites. Rice leaf sheaths of a resistant cultivar were inoculated with the WT or Δrbf1-1 (KO) line harboring TEFp::mCherry, and the number of infection sites showing the mCherry leakage was counted under a fluorescence microscope at 30 hpi. Data are represented as the mean percentages ± SE (n = 5 plants). Student’s t-test was performed on arcsine-transformed data between WT and KO (*, P < 0.05). (B) Images of the 5th leaf blades of the resistant cultivar at 4 days post spray-inoculation. Bar = 5 mm. (PDF) [file ppat.1005921.s021.pdf]
