# Supplementary material for: Magnaporthe oryzae Glycine-Rich Secretion Protein, Rbf1 Critically Participates in Pathogenicity through the Focal Formation of the Biotrophic Interfacial Complex
Source: PLoS Pathog. 2016 Oct 6;12(10):e1005921. doi: 10.1371/journal.ppat.1005921 (PMC5053420; doi:10.1371/journal.ppat.1005921)
Supplement: S9 Fig — The broken lines indicate the hand-sectioned sites shown in Fig 3E. (PDF) [file ppat.1005921.s013.pdf]

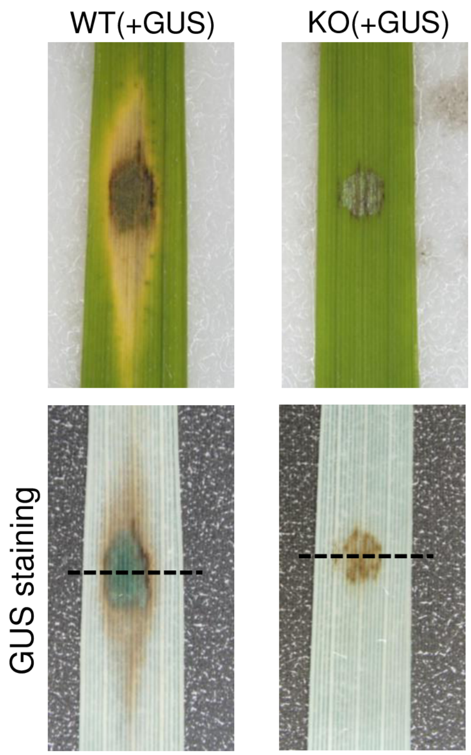

S9 Fig. Symptoms on the rice leaf blades spot-inoculated with the WT- or KO-based transformant and the GUS staining images.
