# Supplementary material for: Magnaporthe oryzae Glycine-Rich Secretion Protein, Rbf1 Critically Participates in Pathogenicity through the Focal Formation of the Biotrophic Interfacial Complex
Source: PLoS Pathog. 2016 Oct 6;12(10):e1005921. doi: 10.1371/journal.ppat.1005921 (PMC5053420; doi:10.1371/journal.ppat.1005921)
Supplement: S10 Fig — (A) qRT-PCR analysis of the expression of NOMT (Os12g0240900), which encodes the key enzyme for sakuranetin biosynthesis, in the inoculated rice leaf blades at 2 dpi. Data are represented as the mean values ± SE of four individual leaves. (B) Quantification of sakuranetin in inoculated leaf blades. Data of five to seven independent extracts in two inoculation assays are represented as mean values ± SE. No significant differences between WT and Δrbf1-1 (KO) were detected using Student’s t-test. Sakuranetin was not detected in the mock-inoculated leaves (n. d.). (PDF) [file ppat.1005921.s014.pdf]

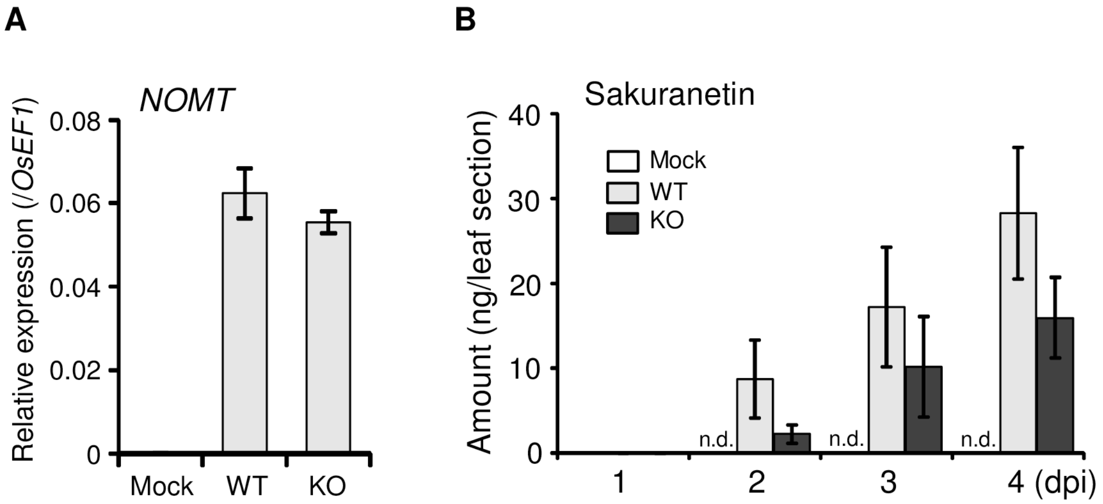

S10 Fig. *RBF1* does not affect the infection-induced production of a flavonoid phytoalexin, sakuranetin.
