# Supplementary material for: Magnaporthe oryzae Glycine-Rich Secretion Protein, Rbf1 Critically Participates in Pathogenicity through the Focal Formation of the Biotrophic Interfacial Complex
Source: PLoS Pathog. 2016 Oct 6;12(10):e1005921. doi: 10.1371/journal.ppat.1005921 (PMC5053420; doi:10.1371/journal.ppat.1005921)
Supplement: S11 Fig — Rice leaf blades were spot-inoculated with a conidial suspension of the WT strain, and total RNA was extracted at 2 dpi for qRT-PCR analysis. Data are represented as the mean values ± SE (n = 4 plants). The expression of OsWRKY45 (Os05g0322900) and SalT (Os01g0348900) was also examined as indicators for SA and ABA signaling, respectively. (PDF) [file ppat.1005921.s015.pdf]

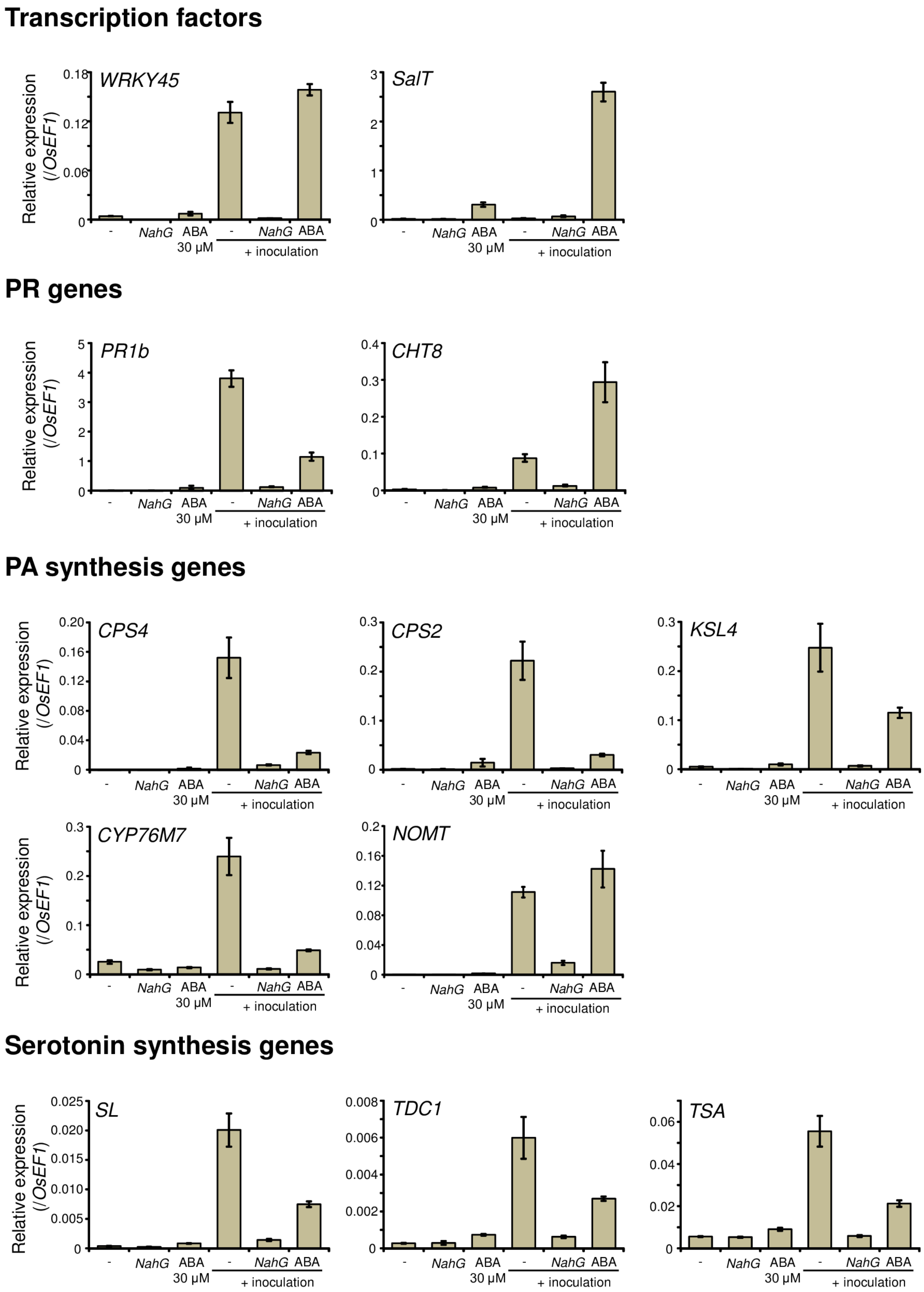

S11 Fig. Activation of defense-related genes by *Magnaporthe oryzae* infection is impaired in *NahG*-expressing and ABA-treated rice.
