# Supplementary material for: Magnaporthe oryzae Glycine-Rich Secretion Protein, Rbf1 Critically Participates in Pathogenicity through the Focal Formation of the Biotrophic Interfacial Complex
Source: PLoS Pathog. 2016 Oct 6;12(10):e1005921. doi: 10.1371/journal.ppat.1005921 (PMC5053420; doi:10.1371/journal.ppat.1005921)
Supplement: S14 Fig — Asterisks, appressoria. Bar = 10 μm. (PDF) [file ppat.1005921.s018.pdf]

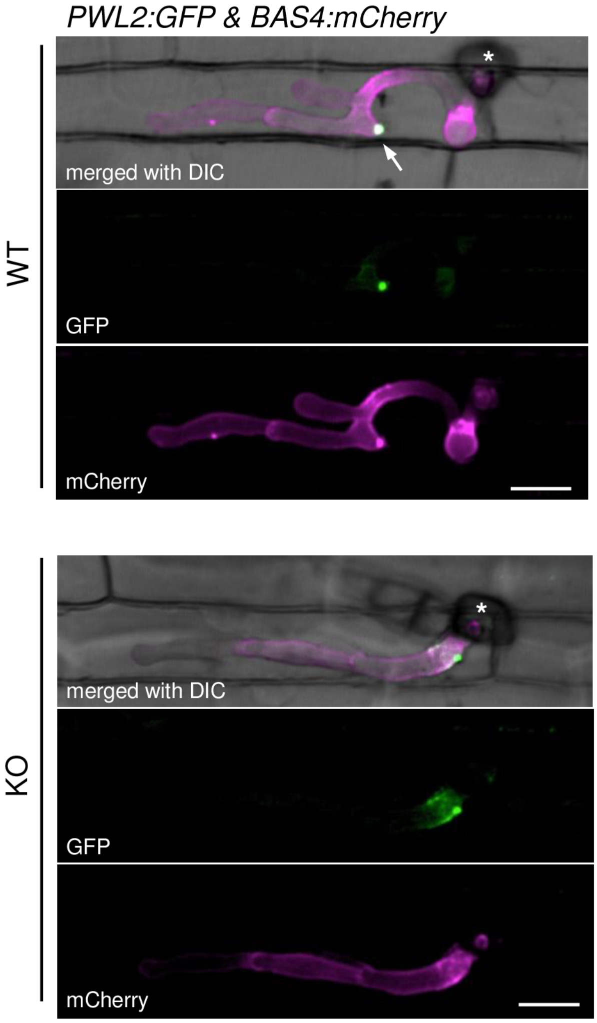

S14 Fig. Confocal images of rice leaf sheath cells infected by the WT or Δ*rbf1-2* (KO) line harboring *PWL2p::PWL2:GFP&BAS4p::BAS4:mCherry* at 36 hpi.
